# Preparation of Surlyn films reinforced with cellulose nanofibres and feasibility of applying the transparent composite films for organic photovoltaic encapsulation

**DOI:** 10.1098/rsos.170792

**Published:** 2017-10-04

**Authors:** Anantaya Lertngim, Manisara Phiriyawirut, Jatuphorn Wootthikanokkhan, Kitti Yuwawech, Weradesh Sangkhun, Pisist Kumnorkaew, Tanyakorn Muangnapoh

**Affiliations:** 1 Department of Tool and Materials Engineering, Faculty of Engineering, King Mongkut's University of Technology Thonburi, Bangkok 10140, Thailand; 2 Division of Materials Technology, School of Energy Environment and Materials, King Mongkut's University of Technology Thonburi, Bangkok 10140, Thailand; 3 Nanotec-KMUTT Center of Excellence on Hybrid Nanomaterials for Alternative Energy, King Mongkut's University of Technology Thonburi, Bangkok 10140, Thailand; 4 National Nanotechnology Center (NANOTEC), National Science and Technology Development Agency (NSTDA), Pathumthani, Thailand

**Keywords:** nanocellulose, encapsulation, organic solar cells, Surlyn

## Abstract

This research concerns the development of Surlyn film reinforced with micro-/nanofibrillated celluloses (MFC) for use as an encapsulant in organic photovoltaic (OPV) cells. The aim of this work was to investigate the effects of fibre types and the mixing methods on the structure–properties of the composite films. Three types of cellulose micro/nanofibrils were prepared: the as-received MFC, the dispersed MFC and the esterified MFC. The fibres were mixed with Surlyn via an extrusion process, using two different mixing methods. It was found that the extent of fibre disintegration and tensile modulus of the composite films prepared by the master-batching process was superior to that of the composite system prepared by the direct mixing method. Using the esterified MFC as a reinforcement, compatibility between polymer and the fibre increased, accompanied with the improvement of the percentage elongation of the Surlyn composite film. The percentage of light transmittance of the Surlyn/MFC films was above 88, regardless of the fibre types and fibre concentrations. The water vapour transmission rate of the Surlyn/esterified MFC film was 65% lower than that of the neat Surlyn film. This contributed to the longer lifetime of the OPV encapsulated with the Surlyn/esterified MFC film.

## 1. Introduction

Solar cell technologies can generally be classified into three main categories: the silicon-based technology, the compound semiconductor-based technology (such as CIGS and CdTe) and the new emerging solar cell technology. The emerging solar cells are characterized by the solution processability and the types of photoactive materials which are normally made from semiconducting polymers, inorganic metal oxide nanoparticles or hybrid organic/inorganic materials. These systems can be referred to as organic photovoltaic cells (OPV), dye-sensitized solar cells (DDSC) and perovskite solar cells (PSC). At the present time, the best global research cell efficiency for DSSC, OPV and PSC, certified by the National Renewable Energy Laboratory (NREL) (https://www.nrel.gov/pv/assets/images/efficiency-chart.png (last retrieved on 29 May 2017)) is 11.9%, 11.5% and 22.1%, respectively.

To upgrade the technology readiness level of these new emerging solar cells, the enhancement of the stability and lifetime of the devices is considered to be one of the most important issues deserving consideration. This is because the power conversion efficiencies of the cells decrease rapidly over time. The performance and stability of these solar cells are known to be highly sensitive to moisture and oxygen. Sharma *et al.* [1], for example, showed that the stability of the OPV cells was strongly affected by the moisture level. After ageing for two weeks in an ambient air atmosphere, the power conversion efficiency (PCE) of the cells dropped rapidly. This was attributed to the realignment of diploes of functional groups in the polymers used for modifying the surface of ITO electrode. Similarly, Glen *et al.* [2] employed PCDTBT:PC70BM as a stable active layer system to investigate the degradation behaviour and degradation mechanism of OPV cells. It was found that the normalized PCE of the cells (ITO/PEDOT:PSS/PCDTBT:PC70BM/Al) dropped rapidly within 4 days under a humid air atmosphere. This was ascribed to the presence of voids and delamination within the device, caused by the reaction of calcium and aluminium in the cathode with water.

To increase the lifetime of these solar cells, several approaches can be used. These include the design of a better device architecture and the adaptation of more stable materials. For example, the work by Glen *et al.* [2] showed that using Ag as a replacement of an Al electrode, degradation was prolonged and the defects (voids) were less apparent. Similarly, Abdulrazzaq *et al.* [3] compared the stability of P3HT:PCBM-based organic solar cells containing different types of hole transport materials, and found that the PANI:CSA-based cells degraded slower than the PEDOT:PSS-based cells. However, the prolonged lifetime of the cell was obtained at the expense of its initial PCE. Alternatively, regardless of the types of electrodes, active layer materials and cell configurations, the lifetime of the new emerging solar cells can be prolonged using an effective encapsulation system. Notably, some encapsulants for OPV cells and organic light emitting diodes (OLED) are commercially available, including the UV curable epoxy-based resins from Ossila (https://www.ossila.com/products/pv-oled-encapsulation-epoxy (last retrieved on 25 May 2017)) and DELO Industrial Adhesives Company [4]. However, the epoxy is a kind of thermoset, and therefore inherently rigid. This might not be suitable for the development and production of flexible solar cells. Besides, the development of high barrier encapsulation materials is still a challenge. For electronic devices, such as OLED and OPV, the ideal water vapour transmission rate (WVTR) and oxygen transmission rate (OTR) values are less than 10^−6^ (g m^−2^ d^−1^) and less than 10^−5^ (cm^3^ m^−2^ d^−2^), respectively [5]. These values are considerably low compared with the inherent OTR and WVTR values of several polymers commonly used in food packaging and sealing applications. For example, the WVTR values of poly(ethylene-co-vinyl alcohol) (EVOH), poly(vinylidene chloride) and poly(ethylene-co-acrylic acid) (Surlyn^®^) are 22–124 (g m^−2^ d^−1^) [6], 0.69 (g m^−2^ d^−1^) (http://www.tappi.org/content/events/07place/papers/paisley.pdf (last retrieved on 25 May 2017)) and 11 (g m^−2^ d^−1^) [7], respectively.

Various strategies have been developed to enhance the barrier properties of polymer encapsulants. US patent no. 8823154 B2 [8] presented the invention of an encapsulation system containing a gas compound, with an epoxy-based spacer layer between one or more barrier films. According to the description of the invention, choices of the barrier films are versatile. These include a low-cost free-standing film commercially available, such as View-Barrier from Mitsubishi and Kapton^TM^. Alternatively, a barrier can have alternating thin films of hybridized sol–gel spin-on glass and polydimethylsiloxane-based and olefin-based elastomers. Seethamraju *et al.* developed EVOH-based films for the encapsulation of organic electronic devices. Using clay (montmorillonite) as a filler, it was found that the WVTR value of the polymer film decreased to 0.0015–0.008 (g m^−2^ d^−1^). Notably, the percentage of visible light transmittance of the composite film also dropped to 60 [7]. To enhance the adhesion properties and processability of the EVOH-based encapsulant, a poly(ethylene-co-acrylic acid) ionomer (known by its trademark name, Surlyn) film was mixed with the EVOH prior to fabricating the film [9]. In this case, WVTR values of the blend films in the range of 0.0023–0.0152 (g m^−2^ d^−1^) were obtained. To further enhance the barrier properties of the EVOH-based film, Madras *et al.* [10] developed a kind of multilayered polymeric composite film, comprising TiO_2_/EVOH interlayer laminated with Surlyn films, for the encapsulation of an organic solar cell (ITO/PEDOT:PSS/P3HT:PCBM/Al). Impressively, it was found that the WVTR value of the neat EVOH/Surlyn films decreased from 1 (g m^−2^ d^−1^) to the range from 2 × 10^−3^ (g m^−2^ d^−1^) to 3 × 10^−5^ (g m^−2^ d^−1^) when the surface-functionalized TiO_2_ nanoparticles were added to the EVOH interlayer. However, the percentage of visible light transmittance of the EVOH/Surlyn-laminated film dropped from 87 to 55 when 2 wt% of the TiO_2_ particles were added. In terms of the lifetime of the organic solar cells, it was found that the normalized PCE of the cells dropped to 0.13–0.18, after an acceleration testing for 120 min. Likewise, Ramamurthy *et al.* [11] applied ZnO nanoparticles to poly(vinyl alcohol) to improve barrier properties of polymer encapsulating film. In this case, WVTR values in the range of 0.008–0.76 (g m^−2^ d^−1^) were achieved. Morlier *et al.* [12] developed multilayered barrier films containing poly(ethylene terephthalate) (PET) substrate, inorganic layers and poly(vinyl alcohol) layers. In this case, a solution of UV curable resin (perhydropolysilazane) was used as a precursor for coating onto the PET. After UV irradiation, the precursor was converted into a solid silica oxide layer. OTR and WVTR values of the barrier films of below 0.06 (cm^3^ m^−2^ d^−1^) and 0.2 (g m^−2^ d^−1^) were claimed, respectively. The normalized PCE of the encapsulated cell over the 450 h of storage time was also greater than that of the normal cell without encapsulation. Bag *et al.* [13] developed a polyisobutylene (PBI)-based encapsulant, capable of curing under UV irradiation in the absence of solvent. The PBI-based encapsulant was claimed to be flexible and compatible with the roll-to-roll printing process. The initial PCE of the encapsulated OPV cell (ITO/PEDOT:PSS/P3HT:PCBM (1/1)/Ca/Al) was 3.05%. After storage in an ambient condition for 20 days, the normalized PCE of the cell dropped to a range of 0.7–0.85, depending on the molecular weight of the polymer. There were, unfortunately, no data reported regarding the barrier and optical properties of the PBI encapsulant. Considerable efforts have also been made to develop encapsulating materials for PSC. These include Surlyn/ethylene-vinyl acetate (EVA) laminate films [14] and a commercial barrier film, ViewBarrier^®^, from Mitsubishi Inc. [15].

Another interesting type of polymer to be developed for use as an encapsulant of solar cells is Surlyn. This is because Surlyn is a kind of ionomer, being inherently transparent, and exhibits good adhesion properties to glass substrate. It has also been used as a sealant for the fabrication of DDSC. The feasibility of using Surlyn nanocomposite films for the encapsulation of organic electronic devices has also been demonstrated by Madras *et al.* [16]. Using the appropriate type and concentration of MgO, a WVTR value of a Surlyn/MgO composite film as low as 0.008 (g m^−2^ d^−1^) can be achieved. The percentage of visible light transmittance of the composite films, ranging 50–82, was also obtained, depending on the metal oxide concentration. After exposure to an accelerated weathering test for 1 h, the lifetime of the encapsulated OPV cells was prolonged.

It would be more useful if the barrier properties of the polymer composite films can be improved without losing a high percentage of visible light transparency. Especially, when the solar cell was encapsulated by some alternative techniques, such as a lamination or a dipping process, prior to cross-linking. In this regard, both sides of the cell would be covered by the encapsulant and thus the transparency of the material plays an important role in controlling the light absorption and performance of the cell. Besides, the use of a transparent encapsulant would allow us to observe the materials on the surface of the cell. As a result, degradation of the solar cell can be studied over time. In this regard, a kind of inherently transparent nanofiller, capable of enhancing barrier properties of the polymer without losing transparency of the film, is desirable. In 2008, Yano *et al.* [17] showed that nanocellulose fibres derived from bacteria can be used as a filler for producing transparent nanocomposites with improved thermal expansion coefficient and mechanical properties. The feasibility of enhancing the mechanical and barrier properties of some polymer/nanocellulose composite films, without sacrificing their transparency, has also been demonstrated. Examples include poly(vinyl alcohol)/nanocellulose [18], EVA/nanocellulose [19], polyurethane/nanocellulose [20] and poly(methyl methacrylate)/cellulose nanocrystal [21] composite systems. In this study, the feasibility of developing composite films, reinforced with micro-/nanofibrillated cellulose, for the encapsulation of OPV via a lamination process was of interest. In this study, it was assumed that by properly controlling the dispersion and distribution of nanocellulose, a kind of tortuous path model, capable of prolonging the diffusion of water and/or oxygen molecules, might be induced [22]. Consequently, lower WVTR and OTR values of the polymer can be expected. In this regard, there are three main technical issues deserving consideration and correction: (i) how to promote the dispersion or disintegration of the micro-/nanofribrillated cellulose (MFC), (ii) how to ensure the even distribution of MFC fibres in the polymer matrix and (iii) how to promote good compatibility between the fibres and the polymer. To cope with the above challenges, in this study, the fibres were pretreated and functionalized with an esterifying agent in order to disintegrate and reduce the hydrophilicity of the fibre. Hexanoyl chloride was used to modify the MFC, taking into account the fact that reactivity towards the esterification of cellulose of the acyl halide is higher and more effective than the carboxylic acid analogue [23]. The aim of this work is to investigate the effects of preparation conditions and surface chemistry on the surface of the MFC on thermo-mechanical, optical and barrier properties of Surlyn/MFC composites. The feasibility of applying the composite films as encapsulants for extending the lifetime of OPV, using a lamination process, was also of interest.

## 2. Material and methods

### 2.1. Materials

Cellulose microfibrils (KY100S) were purchased from the Maido Corporation (Japan). Surlyn-1702 was supplied from the DuPont Company. The density and melt flow index (190°C/2.16 kg) values of the resin were 0.95 g cm^−3^ and 14 g/10 min, respectively. Dimethylformamide (DMF) and acetone AR grade were supplied by RCI Labscan Co. Ltd. Pyridine and hexanoyl chloride were obtained from Sigma-Aldrich Co. Ltd.

### 2.2. Pretreatments and the chemical modification of the micro-/nanofibrillated celluloses

The cellulose microfibres were pretreated by grinding in a mechanical blender before sieving through a 150 µm sieve. They were then dried in an oven at 50°C for 24 h, until reaching a constant weight. The fibres were kept in a desiccator before further use. To modify the chemical structure on the surface of the fibres, the MFC was dispersed in DMF before centrifugation to isolate the cellulose from the solvent. The process was repeated again to ensure that the fibres had disintegrated. After that, the prepared MFC was put into a reaction flask containing pyridine. Five millilitres of hexanoyl chloride was then gradually added to the content in the flask. The reaction was mixed at 110°C for 6 h. After that, it was extracted with acetone in a Soxhlet for 24 h, until neutral pH value was reached.

### 2.3. Preparation and testing of polymer nanocomposites

For comparison, two types of mixing methods were used to prepare the composites. These are the direct mixing method, and the mixing via a preparation of a master-batch. The direct mixing method was carried out using a twin-screw extruder, equipped with a 0.5 mm thick sheet die. Surlyn pellets were mixed with a variety of MFC concentrations (0–1.0 wt%). The temperature profile along the extruder machine, from the feed zone (I), the melting zone (II), the metering zone (III) and the die zone (IV) was 120, 130, 140 and 150°C, respectively. The screw rotating speed used was 40 r.p.m. Finally, the extrudate was passed through a take-off unit, containing a cooling bath and a collecting roll. The extrudate was cut into small pieces and then fabricated into a 0.5 mm thick square film (20 mm × 20 mm), using a hydraulic compression mould operated at 150°C, and a pressure of 1500 Psi (10.34 MPa). The obtained film was then kept in a desiccator at 25°C, at 50% relative humidity (RH).

Alternatively, to promote more disintegration of the fibres, a master-batch containing a high concentration of the fibres (10 wt%) was prepared. In this method, the fibres were first pretreated by dispersing them in water, followed by centrifugal force to isolate the disintegrated fibres from the water. Next, the fibres were re-dispersed in tetrahydrofuran (THF) before mixing with a solution of Surlyn in THF. The mixture was stirred, cast onto a Teflon mould and then dried in an oven at 40°C for 24 h. The dried film was then peeled off and cut into small pieces. Herein, this product is referred to as the master-batch. To obtain the composite film with the desired fibre concentrations (0–3.0 wt%), the prepared master-batch was diluted by mixing it with Surlyn pellets in a twin-screw extruder before converting it into a film by a compression moulding process. Of note, the conditions used for both processes were similar to that which was described above in the case of the direct mixing method.

### 2.4. Characterizations

The Fourier transform infrared (FTIR) spectra of the fibres, both before and after chemical modification, were obtained using a Nicolet iS5 spectrophotometer in an attenuated total reflectance mode. The samples were scanned over a wavenumber ranging from 4000 to 400 cm^−1^, at a resolution of 1 cm^−1^ and 64 scans for each sample. In addition, the extent of esterification was quantitatively determined by a titration technique. One hundred milligrams of the modified MFC was dried in an oven at 100°C and then put into a conical flask containing 40 ml of ethanol (75% aqueous solution). The mixture was stirred at 60°C for 30 min. After that, 40 ml of sodium hydroxide solution (0.1 N) was added to the reaction flask and the content in the flask was stirred for another 15 min. The solution was left for 48 h before undergoing titration. After that, the content in the reaction flask was titrated with a hydrochloric acid solution (0.1 N), using phenolphthalein as an indicator. From the above results, the degree of substitution (DS) was calculated using the following equations:

2.1 $$C(\%)=\left(\frac{(V_{0}-V_{n})\times (0.1N\text{/}1000)\times M_{w}}{\text{cellulose}\;(\text{g})}\right)\times 100$$

and

2.2 $$\text{DS}=\frac{162\times C}{\left[100M_{w}-(M_{W}-1)\times C\right]},$$

where *C* (%) , percentage of esterification; *V*_0_, the total volume of HCl (millilitres); *V_n_*, the volume of HCl used for the titration (millilitres); *M*_w_, formula weight of the hexanoyl chloride; DS, degree of substitution of the esterified cellulose.

Morphology of the MFC and the polymer nanocomposites was examined using a scanning electron microscopy (SEM) instrument (JEOL JSM-6380LV) equipped with a secondary electron detector, a backscattering electron (BE) detector and an X-ray detector. SEM specimen of the fibre was prepared by dropping an aqueous solution (0.1 wt%) of the fibre onto the stub coated with carbon tape. For the polymer composite, the specimens were prepared by cutting the fabricated sheet into rectangular shape and then drying before coating with Au.

Thermal behaviour of the various composite films was examined using differential scanning calorimetry (DSC). The experiment was carried out using DSC 204 Cell/Netzsch Thermal Analysis machine. The sample weight was about 15 mg. The sample was scanned over temperatures ranging between −25°C and 200°C, under nitrogen gas atmosphere at a heating rate of 10^○^C min^−1^. From the DSC thermograms obtained from the second heating scan (heating–cooling and reheating again), percentage crystallinity of the various samples was calculated, using the following equation:

2.3 $$\%\text{Crystallinity}=\frac{\Delta H_{f}}{\Delta H_{f}^{0}(1-W_{f})}\times 100,$$

where Δ*H*_f_, enthalpy of melting of the Surlyn/MFC composites; $\Delta H_{f}^{0}$, enthalpy of melting of the ethylene–acrylic acid copolymer (Surlyn), which is equal to 277.1 J g^−1^ [24]; and *W*_f_, weight fraction of Surlyn in the composites.

### 2.5. Testing of the composite films

The tensile properties of the various Surlyn/MFC nanocomposites were determined using a universal testing machine (Lloyd; LR 50 K). Dumbbell-shaped specimens were prepared by cutting the dried films with a die, in accordance with the ASTM D638 standard. The gauge length used was 50 mm, and the tensile test was carried out at a crosshead speed of 500 mm min^−1^, using a 1 kN load cell. At least five specimens were tested for each sample and the average values of Young's modulus, tensile strength at break and elongation at break were calculated using standard equations. The adhesion properties of the Surlyn films were determined by a peel test, in accordance with the ASTM D3167 standard method. The test was carried out, at room temperature, using a universal testing machine (Lloyd; LR 50 K) operated at a crosshead speed of 100 mm min^−1^. The peeling distance was about 100 mm. The optical properties of the composite films were examined using a Shimadzu UV-3100 spectrophotometer. The sample was cut into a 5 × 5 cm^2^ square-shaped specimen. The UV/visible spectra of various samples were recorded over wavelengths ranging between 200 and 1000 nm. Visible light transmittance was determined in accordance with the ASTM E891-87 standard method. The transmission of light through polymer composite film was integrated over a wavelength range of 400–700 nm.

The barrier property of the polymer nanocomposite films was evaluated by measuring the WVTR and the OTR. The WVTR test was carried out in accordance with the ASTM E96-93 standard method with some modification. Experimentally, the polymer film was cut into a 2 × 2 cm^2^ square piece. After that, the specimen was dried in a vacuum oven at 50°C for 24 h. Next, the dried specimen was placed on top of pre-cut aluminium foil. The aluminium foil mask containing the film was then attached to the top of a conical flask, which was filled with 20 ml of distilled water. The set-up system (the water-filled container and the aluminium foil mask with the film) was weighed and placed in an oven at 40°C/50% RH for 24 h [25]. After that, the system was weighed again. At least three specimens were tested for each sample. The WVTR value was determined using the following equation:

2.4 $${\text{WVTR (g mm m}}^{-2}{\text{d}}^{-1})=\frac{W\times T}{t\times A},$$

where *W*, mass of H_2_O lost from the container (grams); *T*, thickness of the film (millimetres); *t*, the duration for the measurement (day); *A*, the effective area of the film (square metres).

The OTR test was carried out in accordance with ASTM standard 1434-82 (reapproved 2015) at 23°C, and at a controlled RH of 0%, using a GDP-C gas permeability tester. All films were measured in triplicate.

### 2.6. Fabrication, encapsulation and testing of organic photovoltaic cells

The OPV cell with the structure of ITO/PEDOT:PSS/PCDTBT:PCBM/TiO_x_/Al was fabricated on a 2.5 × 2.5 cm^2^ pre-patterned indium tin oxide (ITO) substrate. The substrate was cleaned by a 15 min ultrasonication in distilled water, acetone and isopropanol sequentially. The substrate was then exposed to a 100 W oxygen plasma treatment for 2 min to improve its wetting property. Next, a thin film of PEDOT:PSS solution was deposited atop the ITO substrates by convective deposition with a speed of 3 mm s^−1^ and a deposition volume of 20 µl twice. The PEDOT:PSS film was then dried with a hot plate at 120°C for 30 min. A photoactive layer was deposited via a polymer blend solution. The solution containing 8 mg ml^−1^ of PCDTBT polymer and 32 mg ml^−1^ PC70BM in dichlorobenzene was deposited at a convective speed of 0.75 mm s^−1^ and a polymer volume of 20 µl. The polymer film was left in the air for 10 min before depositing a thin TiO_x_ layer from titanium oxides sol–gel. The TiO_x_ film was deposited with a rate of 1.25 mm s^−1^ with 20 µl solution volume followed by ambient annealing at 80°C for 20 min. Finally, aluminium thin film was deposited as a metal electrode under 5 × 10^−5^ mbar thermal evaporation.

The encapsulation process was carried out using an office-type laminator (PAD3-330C model), in a N_2_-filled glovebox, at a RH value of below 30% and at an oxygen level of 0 ppm. The actual lamination temperature was 140°C and the lamination process was repeated for five cycles. The residence time for each lamination cycle was 10 s. *J*–*V* characteristics of the various cells were measured as a function of storage time using a Keithley 2400 Source Meter under 1.5 AM. The light source was generated by a solar simulator (Newport 91150 V model, 1000 W, xenon lamp), equipped with a 1.5 G air mass filter. The light was directed at the bottom side of the cell. The active area of the OPV cell was 0.1 cm^2^. The averaged *V*_oc_, *I*_sc_, fill factor (FF) and PCE values, representing three replicated cells for each OPV device, were then calculated and reported.

## 3. Results and discussion

**Figure 1.**
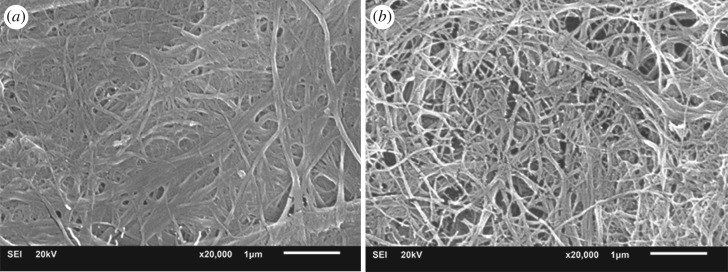
SEM images of the MFC (*a*) and the modified MFC (*b*).

Figure 1 shows SEM images of the MFC, both before and after experiencing chemical modification with hexanoyl chloride. The average fibre diameter values of the MFC and the esterified MFC, determined using an ImageJ analysis, were 0.07 (±0.02) µm and 0.06 (±0.01) µm, respectively. Notably, accumulation and entanglement of the fibres decreased after treating them with hexanoyl chloride. The above effects can be ascribed to the change in chemical structure on the surface of the fibres after the chemical reaction. This was, in turn, altering the interaction between fibres. After chemical interaction, hydrogen bonding between hydroxyl groups on the surface of the fibres was suppressed due to the fact that some of these groups had been converted into ester groups which were less polar.

**Figure 2.**
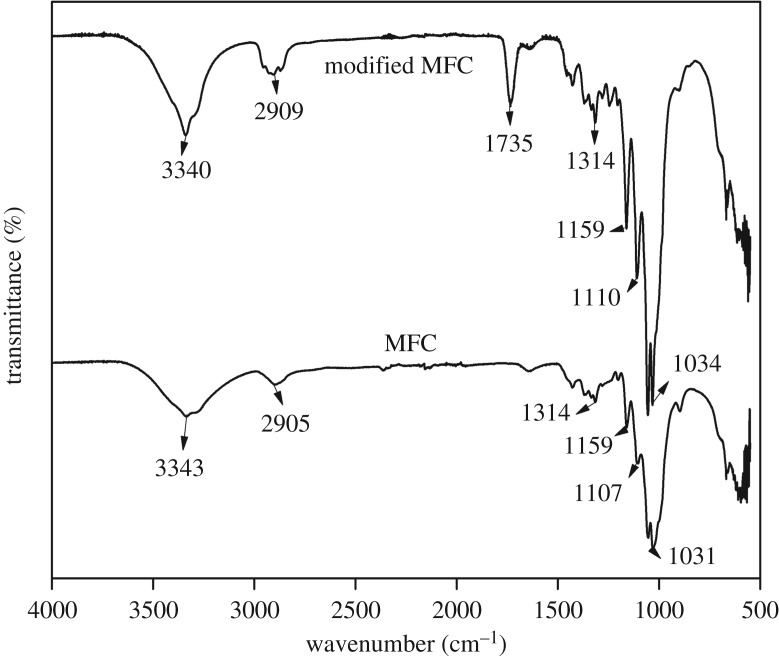
FTIR spectra of the normal MFC and the modified MFC.

Figure 2 shows the FTIR spectra of MFC, both before and after experiencing the chemical modification with hexanoyl chloride. The FTIR spectrum of the unmodified MFC shows the presence of absorption peaks at 3340, 2909, 1314 and 1159 cm^−1^, representing the vibration of O–H (stretching), C–H (stretching), CH_2_ rocking and C–O–C (asymmetric stretching), respectively [26]. After reacting with hexanoyl chloride, a new peak at 1735 cm^−1^ emerged. This can be ascribed to the vibration of a C=O bond in the carbonyl group of the esterified. A similar spectral change has also been observed by Menezes *et al.* [27] in a study on polyethylene nanocomposite reinforced with an esterified cellulose whisker. In our present study, the DS of the esterified MFC, determined using a titration technique, was 0.582 (±0.05). The value is slightly lower than that reported by Menezes *et al.* [27]. The discrepancy can be due to the different reaction conditions, different geometry and different surface area of the nanocellulose used (2 g of cellulose whisker reacting with 5.2 ml of hexnoyl chloride for 4 h in the literature work, and 3 g of cellulose microfibres reacting with 5 ml of hexanoyl chloride for 6 h in this study).

**Figure 3.**
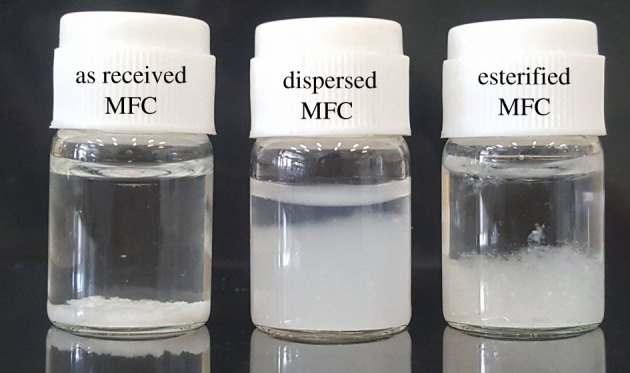
Photographs of aqueous suspensions of different types of cellulose.

Changes in polarity and the extent of disintegration of the fibres after the chemical modification are also reflected by their appearance in a water suspension. Figure 3 shows photographs of the various types of MFC suspended in water. The as-received MFC (without any pretreatment) is clearly precipitated in the water. On the other hand, the middle bottle containing MFC fibres, which were pretreated by dispersing them in the water, are more opaque and less phase separated. This implies that the fibres have disintegrated. After experiencing the chemical modification, the esterified MFC fibres were isolated from the water again. This was due to the change in the chemical structure on the surface of the fibres, from hydroxyl groups to hexanoyl groups, which are less hydrophilic. The extent of phase separation was, however, incomplete due to a steric effect provided by the hexanoyl side chains and the fact that the fibres had disintegrated by dispersion in water.

### 3.1. Effects of mixing methods on structure–properties of Surlyn/micro-/nanofibrillated celluloses films

**Figure 4.**
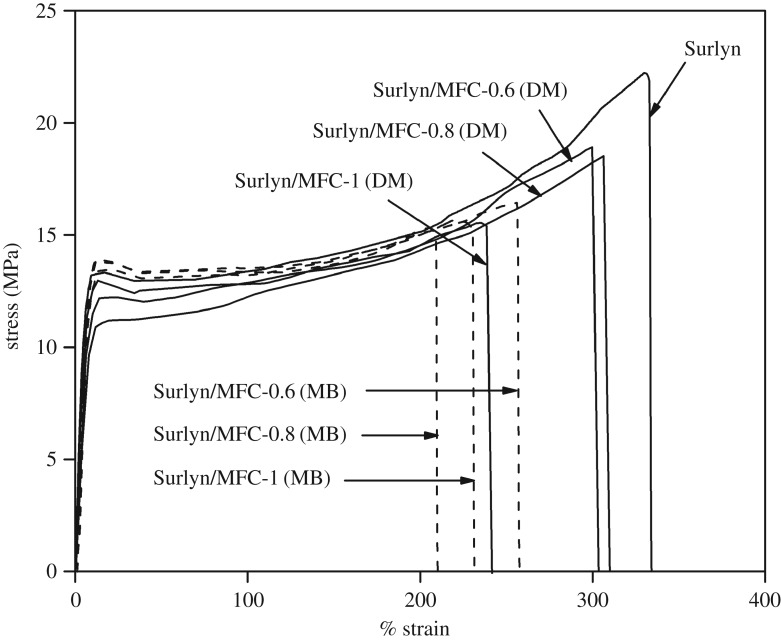
The overlaid stress–strain curves of Surlyn/MFC composite films, prepared using different mixing methods and fibre concentrations.

Figure 4 shows typical stress–strain curves of both the neat Surlyn film and the Surlyn/MFC composite films, prepared using different mixing methods. In general, it can be seen that the stress–strain profiles of the polymer specimens changed remarkably with the presence of MFC. It seems that the composite films became weaker and less ductile than the neat Surlyn film, taking into account the lower yield stress, the lower ultimate stress, and the lower percentage strain of the stress–strain profiles. It was also noteworthy that the above effects became more pronounced when the composites were prepared via the master-batch process. The above results indicate that the mechanical properties of the system were strongly affected by the mixing method used.

**Figure 5.**
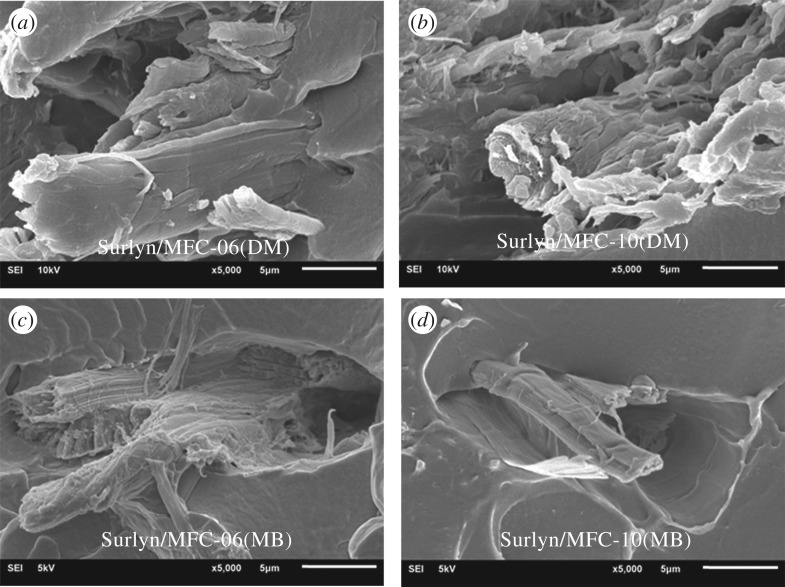
SEM images of Surlyn/MFC composites, prepared by a direct mixing (DM; *a*,*b*) and using a master-batch (MB; *c*,*d*) method.

Tensile properties of the various films, described in terms of the average tensile strength, the average tensile modulus and the average percentage elongation, were calculated and are summarized in table 1. Again, it can be seen that the strength and elongation values of the Surlyn/MFC films were lower than those of the neat Surlyn film, regardless of the mixing method and fibre concentrations. Consideration of the SEM images of the composite films (figure 5) reveals the presence of gaps at the interface between fibres and the matrix. This contributed to the decrease of tensile strength and percentage elongation values of the Surlyn film after the addition of MFC.

**Table 1.** Tensile properties of the various Surlyn/MFC composite films.

			tensile properties		
sample names	mixing method	MFC content (%)	strength (MPa)	modulus (MPa)	elongation (%)
Surlyn	n.a.	0	21.72 (±1.66)	292.26 (±13.83)	349.54 (±5.22)
Surlyn/MFC-06 (DM)	direct mixing	0.6	18.36 (±1.4)	236.41 (±5.20)	292.64 (±24.77)
Surlyn/MFC-08 (DM)		0.8	18.01 (±1.0)	230.54 (±10.02)	311.16 (±4.58)
Surlyn/MFC-10 (DM)		1.0	15.25 (±1.51)	244.11 (±5.31)	224.30 (±29.57)
Surlyn/MFC-06 (MB)	master-batching	0.6	16.97 (±1.26)	293.72 (±13.90)	254.66 (±8.77)
Surlyn/MFC-08 (MB)		0.8	15.28 (±0.496)	282.99 (±14.238)	214.21 (±18.547)
Surlyn/MFC-10 (MB)		1.0	15.33 (±0.387)	298.39 (±15.823)	237.10 (±7.210)

In this study, the effects of fibre concentration on the tensile properties of the composite films were not remarkable, taking into account the standard deviation values. Especially, tensile strength and tensile modulus values of the composites containing 0.6 and 0.8 wt% MFC are not different, provided that the same mixing method was used. However, it was found that the effect of the mixing method on the tensile properties of the composite films is significant. Surprisingly, the tensile modulus values of the composite films, prepared using a direct mixing method, were lower than those of the neat Surlyn film. This was not the case when the composites were prepared by the master-batching process. The above effect was different from that which was observed when nano-MgO particles were added to the polymer [16]. The discrepancy can be attributed to the fact that nano-MgO and cellulose are different in terms of chemical nature and dimensions. The smaller size of the particles generally results in a better dispersion.

In this study, the results from the DSC thermal analysis technique, summarized in table 2 (see also the electronic supplementary material), reveal that the percentage of crystallinity of the various composite films was not significantly different. However, it is noteworthy that the morphology of the fibres was different when the mixing method was changed (figure 5). The fibres in the composites, prepared by the master-batching technique, were more disintegrated compared to the fibres in the system prepared by direct mixing. This contributed to the greater tensile modulus values of the former composite systems.

**Table 2.** Thermal properties of Surlyn composite films reinforced with different types and concentrations of MFC, using two different mixing methods.

	thermal properties			
	direct mixing		master-batching	
MFC content (%)	*T*_m_ (°C)	*X*_c_ (%)	*T*_m_ (°C)	*X*_c_ (%)
0	93.1	17.81	93.1	17.81
0.6	93.4	16.45	92.4	17.30
0.8	93.1	15.58	93.5	17.08
0.8^a^	n.a.	n.a.	92.2	16.88
1.0	93.7	17.44	92.4	17.09
1.0^a^	n.a.	n.a.	93.6	17.34
3.0	92.5	18.07	92.1	17.88
3.0^a^	n.a.	n.a.	93.0	17.84

^a^The composite films reinforced with the modified MFC.

**Figure 6.**
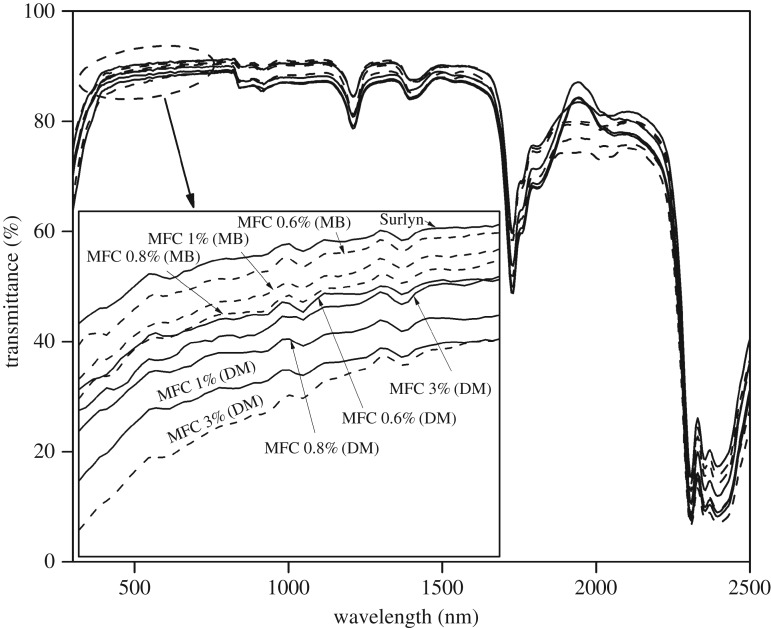
UV/visible spectra of various Surlyn films containing different concentrations of MFC and prepared using two different mixing techniques.

Figure 6 shows the UV/visible spectra of the various composite films. The percentage transmittance in the visible region was calculated and is summarized in table 3. The light transmittance through the neat Surlyn film was about 90%. When the MFC was directly added to the polymer, the transmittance values slightly decreased to the range of 87.7% and 89.18%, depending on the fibre concentration. For the Surlyn composite films containing 0.6 and 0.8 wt% MFC, the percentage light transmittance was not significantly different. However, as the MFC concentration was increased to 1.0 wt%, the transmittance value decreased to 87.7%. Notably, when the MFC was applied to the Surlyn film by a master-batching method, the percentage of light transmittance of the film hardly changed. We explain the above effect in the light of different morphology of the composite systems prepared using different mixing methods. Nevertheless, regardless of the mixing method and fibre concentration, the overall light transmittance of the composite films was above 85% which is sufficient for solar cell encapsulation application [28,29].

**Table 3.** Percentages of visible light transmittance and water vapour transmission rate of the Surlyn/MFC composite films.

sample names	mixing method	MFC content (%)	transmittance (%)	WVTR (g mm (d^−1^ m^−2^))
Surlyn	n.a.	0	90.39 (±0.14)	11.62 (±0.11)
Surlyn/MFC-06 (DM)	direct mixing	0.6	89.18 (±0.07)	9.86 (±1.63)
Surlyn/MFC-08 (DM)		0.8	88.42 (±0.38)	11.1 (±1.76)
Surlyn/MFC-10 (DM)		1.0	87.76 (±0.23)	6.74 (±1.55)
Surlyn/MFC-06 (MB)	master-batching	0.6	90.08 (±0.04)	7.03 (±0.54)
Surlyn/MFC-08 (MB)		0.8	89.31 (±0.16)	6.25 (±0.41)
Surlyn/MFC-10 (MB)		1.0	89.60 (±0.14)	6.12 (±0.10)

In terms of barrier properties, the results from table 3 also show that the WVTR of Surlyn film dropped after mixing with the MFC. The WVTR values of the composite films prepared using a master-batching method were also lower than those prepared by the direct mixing method. The difference was attributed to the different levels of fibre disintegration and the different morphology of the composites (figure 5).

### 3.2. Structure–properties of Surlyn films reinforced with the modified micro-/nanofibrillated celluloses

**Figure 7.**
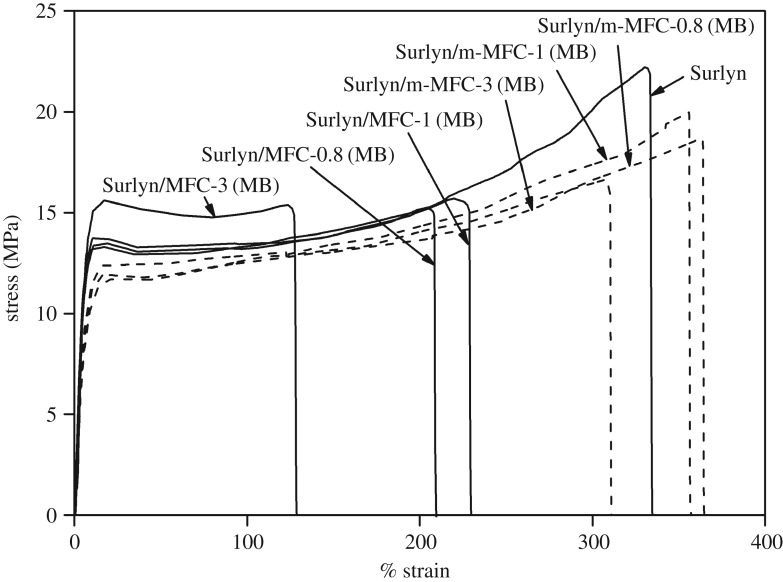
The overlaid stress–strain curves of Surlyn/MFC composite films, reinforced with different types and concentrations of MFC.

**Figure 8.**
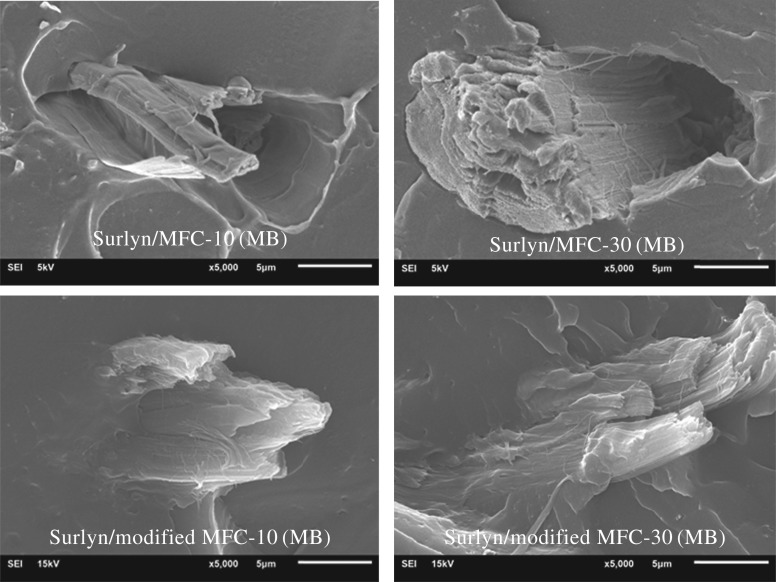
SEM images of Surlyn films reinforced with different types and concentrations of MFC.

Figure 7 shows stress–strain curves of a Surlyn film, and the Surlyn composite films reinforced with different types and concentrations of MFC. The tensile properties of the various composites were also calculated and are summarized in table 4. It can be seen that the tensile strength of the Surlyn composite films, reinforced with the modified MFC, is greater than that of the Surlyn film reinforced with the unmodified MFC. This implies that the modified fibres were more compatible with Surlyn. The above notion was confirmed by considering SEM images of the composite films (figure 8). Better adhesion between polymer matrix and the fibres can be observed when the chemically modified fibers were used. This was ascribed to the presence of hexanoate groups on the surface of the esterified fibres which are less polar than the hydroxyl groups of the neat MFC. The above change favours the interaction between ethylene–(meth)acrylic acid copolymer and the fibres.

**Table 4.** Tensile strength, modulus, elongation and peel strength of the Surlyn composite films reinforced with different types and concentrations of MFC.

			tensile properties			
sample names	fibre types	content (%)	strength (MPa)	modulus (MPa)	elongation (%)	peel strength (N mm^−1^)
Surlyn	n.a.	0	21.72 (±1.66)	292.26 (±13.83)	349.54 (±5.22)	0.19 (±0.037)
Surlyn/MFC-08 (MB)	normal	0.8	15.28 (±0.496)	282.99 (±14.238)	214.21 (±18.547)	0.22 (±0.019)
Surlyn/MFC-10 (MB)		1.0	15.33 (±0.387)	298.39 (±15.823)	237.10 (±7.210)	0.21 (±0.028)
Surlyn/MFC-30 (MB)		3.0	15.76 (±0.166)	352.58 (±3.583)	138.45 (±14.734)	0.21 (±0.026)
Surlyn/m-MFC-08 (MB)	esterified	0.8	18.25 (±0.327)	282.99 (±14.238)	364.43 (±1.695)	0.26 (±0.025)
Surlyn/m-MFC-10 (MB)		1.0	18.28 (±0.510)	298.39 (±15.823)	351.04 (±22.894)	0.23 (±0.010)
Surlyn/m-MFC-30 (MB)		3.0	16.41 (±1.008)	282.99 (±14.238)	304.09 (±24.745)	0.24 (±0.017)

Notably, by comparing the tensile properties of the neat Surlyn film (without fibres), the percentage elongation of the Surlyn composite film, reinforced with 0.8 wt% modified MFC, was higher, whereas its modulus and tensile strength values were lower. In other words, the composite films became softer and more elongated. It was apparent that the esterified MFC acted as a kind of plasticizer, inducing more flexibility of the ethylene–acrylic acid copolymer (the so-called Surlyn film herein) chains. Work by Wakabayashi *et al.* [30] showed that owing to the similar chemical structures between the metal soap and the Surlyn ionomer, some metal soaps are miscible with ethylene–(meth)acrylic acid ionomer. This led to a plasticizing effect of the polymer. In relation to this study, it was postulated that hexanoate groups on the surface of the esterified MFC are also miscible with the ionic aggregates in the Surlyn ionomer. This weakened the ionic interaction between (meth)acrylic acid units in the copolymer molecules. As a result, a plasticizing effect was induced. However, as the concentration of modified MFC used for compounding with Surlyn was increased above 0.8 wt%, the plasticizing effect disappeared. It was believed that this could be due to some agglomerations of the fibres, which were overridden by the interaction between the polymer and the fibres.

**Figure 9.**
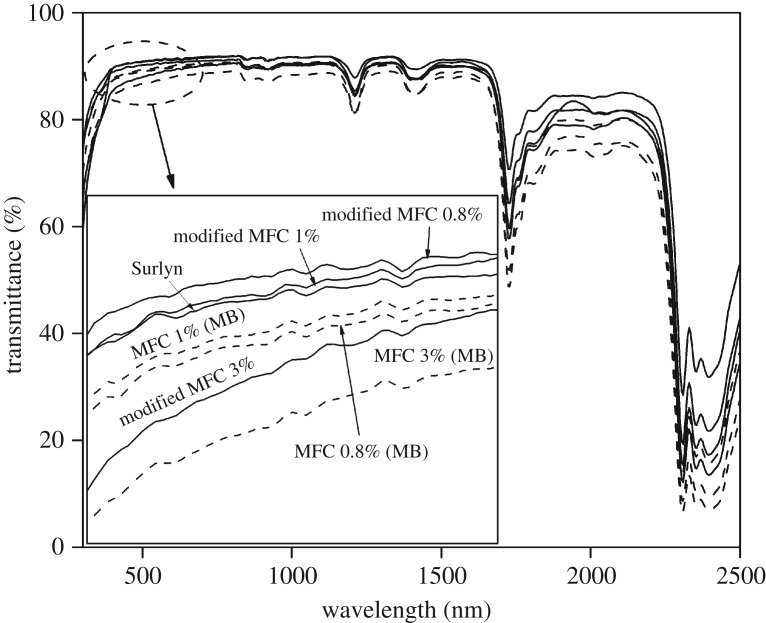
UV/visible spectra of Surlyn films containing different types and concentrations of MFC.

Eventually, the developed Surlyn composite films were to be applied for the encapsulation of OPV cells through a lamination process. In this regard, attempts were also made to evaluate the adhesion property of the composites. The strength of adhesion of the film on to glass substrate was determined by a peel test. The results from table 4 show that the peel strength of the laminate slightly increased from 0.19 N mm^−1^ to the range of 0.21–0.26 N mm^−1^, depending on the fibre type and fibre concentration. The above increase was attributed to the greater cohesive strength of the Surlyn composite films due to a reinforcing effect of the fibres. This also implies that the polymer is still well adhered to the glass substrate. In other words, the presence of small concentrations (0.8–3.0 wt%) of the fibres did not have an adverse effect on the adhesion property of the Surlyn films. Figure 9 shows UV/visible spectra of the Surlyn composite films reinforced with different types and concentrations of MFC. The percentage of visible light transmittance of the various films was also calculated and is summarized in table 5. Overall, the percentage of visible light transmittance of the Surlyn/modified MFC films ranged from 88.5 to 90.1, depending on the fibre concentration. These values are slightly lower than those of the neat Surlyn film (without any fibres, 90.4%). The lowest percentage of visible light transmittance was observed when 3.0 wt% of the modified MFC was applied. Nevertheless, the transparency of the composite films is sufficiently high with respect to the general specifications of encapsulating materials for solar cell industries [28,29]. Table 5 shows the results obtained from the WVTR test. The WVTR value of the neat Surlyn film obtained from this study was 11.62 (g m^−2^ d^−1^) which is fairly close to that reported by the supplier (11 g m^−2^ d^−1^, for a 2 mm thick film) [31]. When the modified MFC was applied to the polymer, the WVTR values of the composite films obviously dropped. In this study, the lowest WVTR value obtained was 3.99 (g m^−2^ d^−1^). This corresponds to the system containing 1.0 wt% of the modified MFC. Beyond this fibre concentration level, the WVTR value tended to increase again, probably due to an agglomeration of the fibres. It is also noteworthy that the WVTR values of the Surlyn/modified MFC composite films are lower compared to those of the Surlyn/MFC analogues, provided that the same fibre concentration is considered and compared. Similarly, the OTR values of the Surlyn films reinforced with the modified MFC significantly decreased compared to those of the neat Surlyn film (see also the electronic supplementary material). This was not the case for the Surlyn/MFC series, taking into account the standard deviation values. The discrepancy can be attributed to better fibre disintegration and better compatibility between fibres and polymer.

**Table 5.** Percentages of visible light transmittance and water vapour transmission rates of the Surlyn composite films reinforced with different types and concentrations of MFC.

sample names	fibre types	content (%)	transmittance (%)	WVTR (g mm (d^−1^ m^−2^))	OTR (cm^3^ (m^−2^ d^−1^ bar^−1^))
Surlyn	n.a.	0	90.4 (±0.14)	11.62 (±0.11)	287 (±18.90)
Surlyn/MFC-08 (MB)	normal	0.8	89.3 (±0.16)	6.25 (±0.41)	268 (±14.57)
Surlyn/MFC-10 (MB)		1.0	89.6 (±0.14)	6.12 (±0.10)	323 (±25.00)
Surlyn/MFC-30 (MB)		3.0	87.2 (±0.13)	10.74 (±1.65)	268 (±21.07)
Surlyn/m-MFC-08 (MB)	esterified	0.8	90.9 (±0.13)	5.92 (±0.21)	232 (±19.55)
Surlyn/m-MFC-10 (MB)		1.0	90.6 (±0.15)	3.99 (±0.58)	247 (±14.84)
Surlyn/m-MFC-30 (MB)		3.0	88.6 (±0.16)	4.95 (±0.07)	253 (±7.23)

Considering the above results, and taking into account the compromised tensile modulus, peel strength, transparency, WVTR and OTR values, the Surlyn composite film, reinforced with 0.8% of the modified MFC, was selected for further use to encapsulate OPV cells. In addition, for comparative purposes, the neat Surlyn film (without any fibres) and the Surlyn film reinforced with the normal MFC (without any chemical modification) were also selected for encapsulation and a further study on the performance and durability of OPV cells.

**Figure 10.**
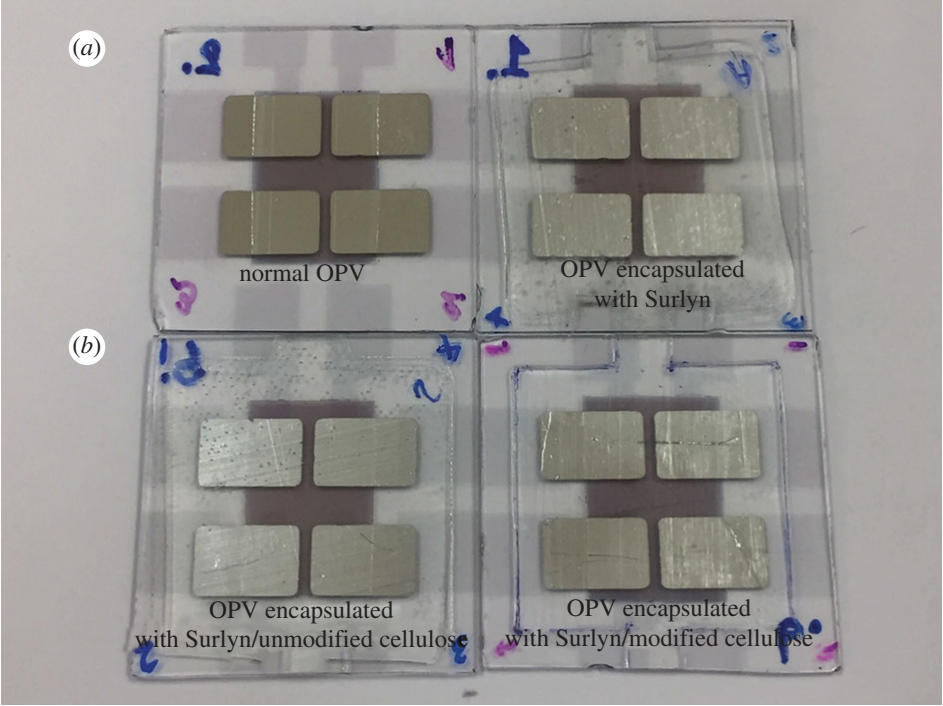
Photographs of the OPV cells with and without encapsulation; at the front side (*a*) and the back side (*b*).

Figure 10 shows the appearance of the OPV cells, with and without encapsulation. Note that the encapsulant materials remain clear in the cell active area. There was no bubble, void or wrinkle observed after the lamination. This implies a possibility of obtaining the proper cells.

**Figure 11.**
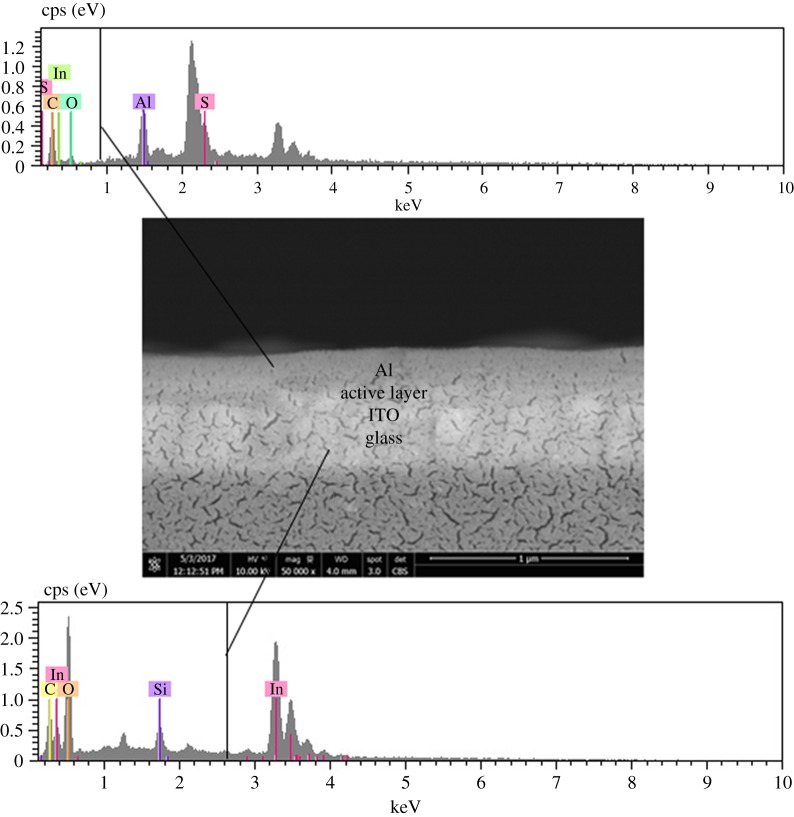
BE SEM image and EDX patterns from different layers of the cross-sectioned OPV cell.

Figure 11 shows the BE SEM image and energy-dispersive X-ray (EDX) patterns of the cross-sectioned OPV cells, with and without encapsulation. The BE image of the bare OPV cell shows the presence of multilayers, representing the materials used for fabricating the cells. Using an EDX technique, Si, In and Al elements representing the glass substrate, ITO and the top Al electrode, can be identified, respectively.

### 3.3. Photovoltaic performance and durability of the organic photovoltaic

**Figure 12.**
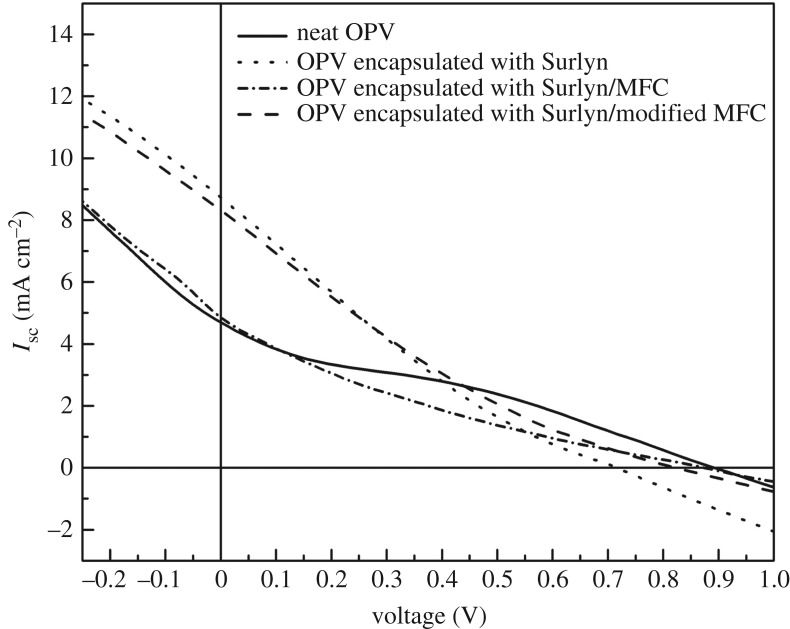
*I*–*V* curves of the various OPV cells.

**Figure 13.**
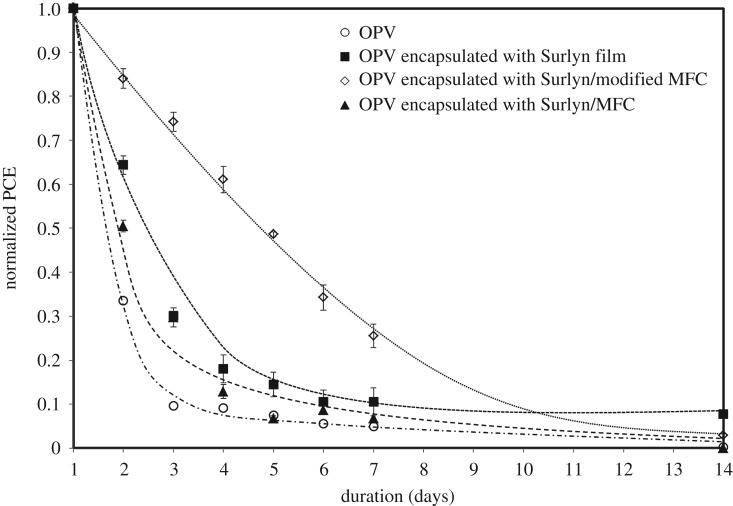
Changes in the normalized power conversion efficiency of the OPV cells, encapsulated with different types of Surlyn films.

Last but not least, the photovoltaic performance and durability of the OPV cells deserve consideration. Figure 12 shows typical *I*–*V* curves of the various OPV cells. The PCE values and the related parameters (open circuit voltage, *V*_oc_; short circuit current, *I*_sc_; FF) of each cell were also calculated and are summarized in table 6. In this study, the maximum PCE of the OPV cell obtained was 1.27%. We realize that this value is low compared to some reports in the literature. In our opinion, the discrepancies could be attributed to the variation of layer thickness, active area and types of active layer and electrode materials used. Figure 13 shows changes in normalized PCE values of various OPV cells. The normalized PCE value of the bare OPV cell (without any encapsulation) dropped rapidly within a couple of days. This was not the case when the OPV cell was encapsulated with the normal Surlyn film (without any cellulose fibres). However, when the Surlyn/MFC was used as an encapsulant, the normalized PCE of the cell dropped rapidly. The above effect could be ascribed to the presence of some defects within the composite film, allowing some migration and ingression of moisture through the cell underneath. On the other hand, when the Surlyn composite film reinforced with 0.8 wt% of the esterified MFC was used, the lifetime of the OPV cell was the most prolonged. After 5 days, a normalized PCE value of about 0.5 could be maintained. This reflects a better compatibility between the fibres and the polymer matrix, minimizing some voids, gaps and other defects. However, in this study, the long-term stability of the encapsulated OPV cells is not satisfactory. After 4 days, the normalized PCE value dropped to 0.17. Even though this value is higher than that of the bare OPV cell, the above results suggest that the Surlyn-based encapsulants and the encapsulation technology of OPV cells have to be further improved.

**Table 6.** Open circuit voltage (*V*_oc_), short circuit current (*I*_sc_), fill factor (FF) and power conversion efficiency (PCE) of the various OPV cells.

devices	*V*_oc_ (mV)	*I*_sc_ (mA cm^−2^)	FF (%)	PCE (%)
OPV (without encapsulation)	888.31	4.64	30.82	1.27
OPV with neat Surlyn	610.7	7.06	23.56	1.04
OPV with Surlyn/MFC	667.2	4.43	20.17	0.63
OPV with Surlyn/m-MFC	663.5	5.64	23.26	0.95

**Figure 14.**
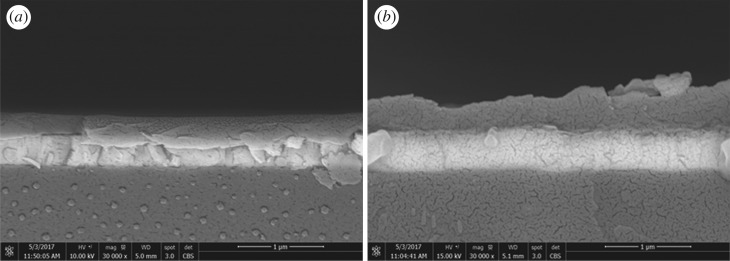
BE SEM images of the OPV cell encapsulated with the neat Surlyn film (*a*), and encapsulated with the Surlyn/modified MFC composite film (*b*).

**Figure 15.**
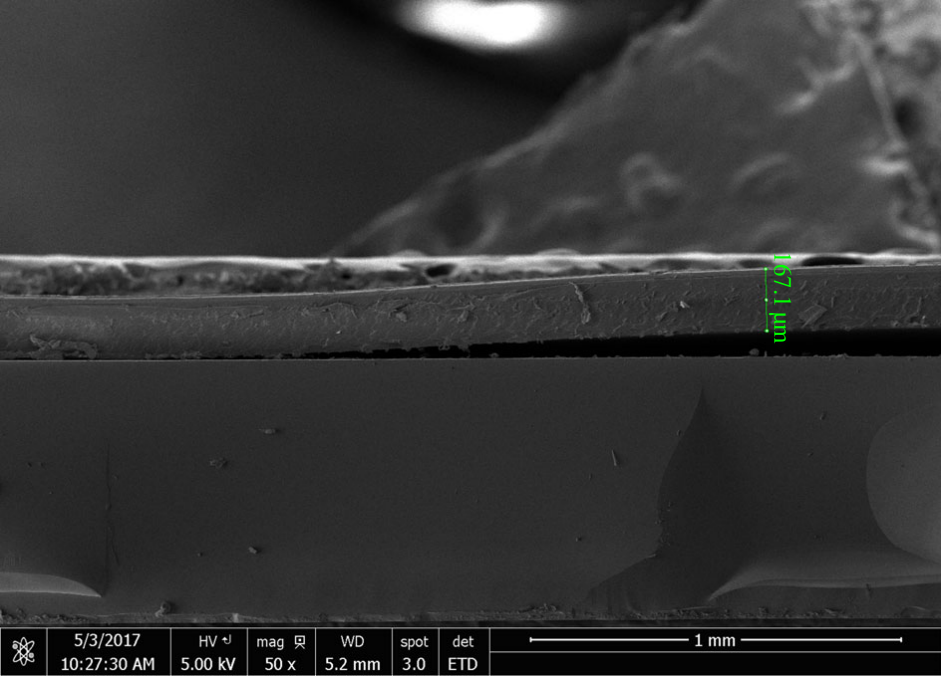
SEM image of a cross-sectioned OPV encapsulated with the Surlyn/modified MFC film.

Figure 14 shows BE SEM images and morphology of the encapsulated cells. Some slivers, fragmentation and blistering in the layers of the cells were noted. This could be due to some stress and heat imposed on the layers underneath the cells during the lamination process. Furthermore, consideration of the low magnification SEM image of the cross-sectioned cells encapsulated with Surlyn/modified MFC film reveals the presence of gaps and delamination between the Surlyn film and the Al electrode (figure 15).

The above delamination and defects could be attributed to the encapsulation process and the actual conditions used for applying the Surlyn film onto the OPV cells. In this study, rather than using a heat gun to melt the Surlyn film for lamination bonding, the Surlyn-based films were directly applied to the cells using an office-type lamination process. Note that this process is a kind of continuous process and not a static process. In this regard, it was possible that the bonding time, bonding pressure and the bonding temperature during the lamination process were not sufficiently high enough to induce a complete melting and wetting of the Surlyn film on the substrate (the OPV cells). According to a technical data sheet of the Surlyn (1702) provided by the supplier (http://www.dupont.com/content/dam/dupont/products-and-services/packaging-materials-and-solutions/packaging-materials-and-solutions-landing/documents/surlyn_1702_1.pdf) [31], the melting temperature of the resin is 94°C, whereas the recommended temperatures for the extrusion and lamination processes range from 160°C (in the feed zone) up to 235°C (in the die zone). In this study, however, the actual temperature during the lamination process was 140°C, and the total residence time for lamination was 50 s. No further attempt was made to increase the temperature and number of passes in the lamination process, because thermal and mechanical stress could destroy the active layer underneath the electrodes. In bottom lines, due to the presence of the above defects, some moisture and oxygen can penetrate into the cells. This contributes to the decrease of the normalized PCE of the encapsulated OPV over time. It seems that the lamination process for the composite and the OPV device substrate has to be optimized in order to ensure the minimal external effects during the study. One possible strategy is to use a hot tack for sealing the cells by heat, at the edges without disturbing the device performance. Alternatively, an appropriate adhesive, such as an epoxy glue, might be applied, similar to that reported by Madras *et al.* [16]. Last but not least, even though the lowest WVTR value of Surlyn/MFC composite film obtained from this study is still far from the ideal range required for use with organic electronic devices, the achievement in terms of improved barrier properties is considered useful for other relevant applications such as encapsulation for food packaging and for silicon-type photovoltaic cells. Especially, the concept of using MFC as a filler to improve barrier properties of polymers might be extended to other systems commonly used for the encapsulation of silicon photovoltaic cells such as EVA copolymer and poly(vinyl butyral).

## 4. Conclusion

Overall, this study has demonstrated that the mechanical and barrier properties of Surlyn film can be effectively enhanced by reinforcing it with esterified MFC, provided that the mixing method and fibre content are properly controlled and optimized. Regardless of the fibre types and fibre concentrations, a percentage visible light transmittance of the Surlyn/MFC films above 88% could be maintained. It was also possible to apply the Surlyn composite film as an encapsulant for OPV cells. The use of Surlyn/modified MFC as an encapsulating material can prolong the lifetime of the OPV cell for up to 7 days. For a longer lasting cell, however, the conditions and techniques for bonding the Surlyn film with the OPV cell have to be further improved and optimized

## Supplementary Material

DSC thermograms of the Surlyn-MFC composite films

## Supplementary Material

OTR results

## Data accessibility

The datasets supporting this article have been uploaded as part of the electronic supplementary material.
