# Supplementary material for: Preparation of Surlyn films reinforced with cellulose nanofibres and feasibility of applying the transparent composite films for organic photovoltaic encapsulation
Source: R Soc Open Sci. 2017 Oct 4;4(10):170792. doi: 10.1098/rsos.170792 (PMC5666266; doi:10.1098/rsos.170792)

Surlyn film#1

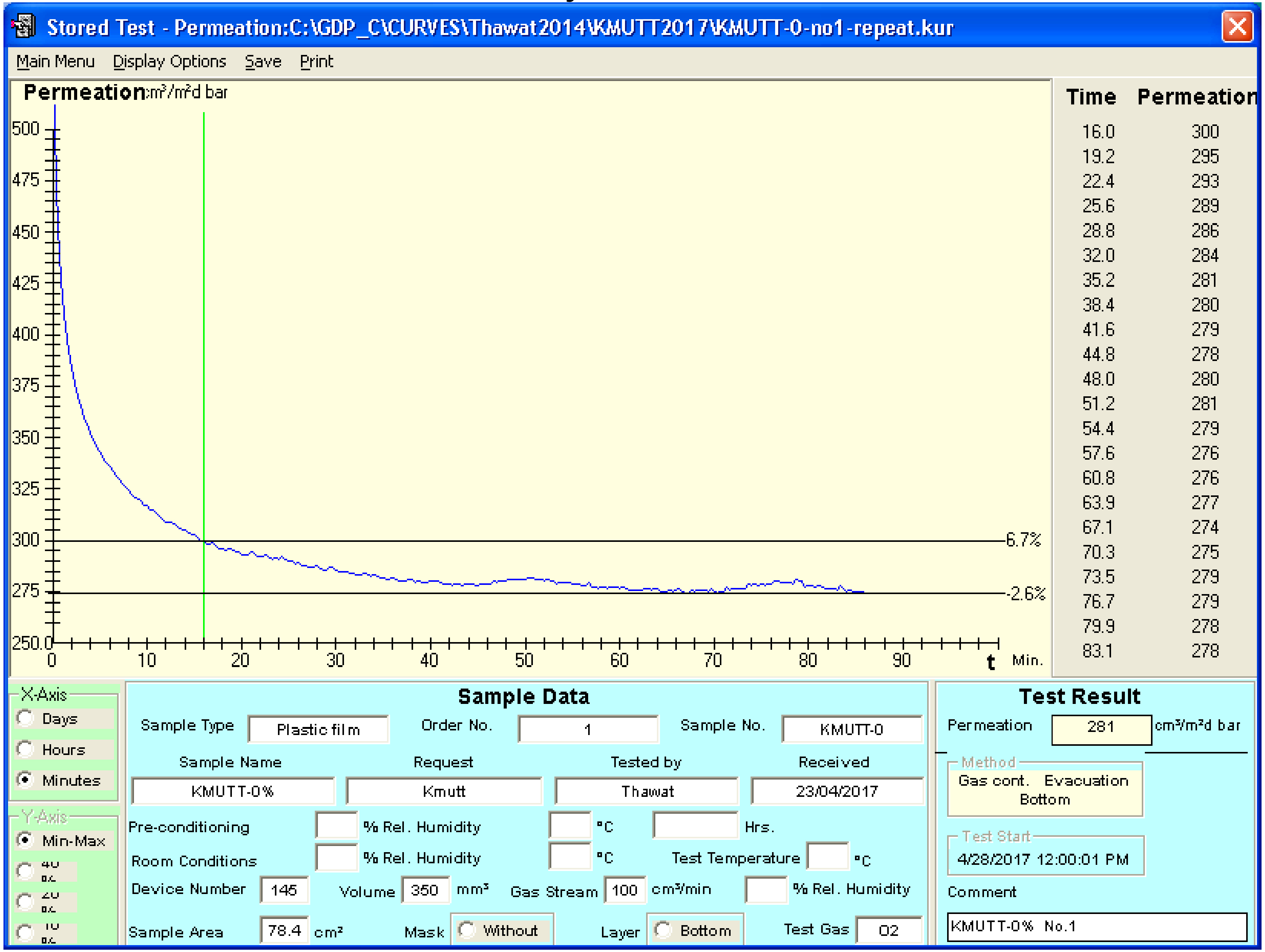

Surlyn film#2

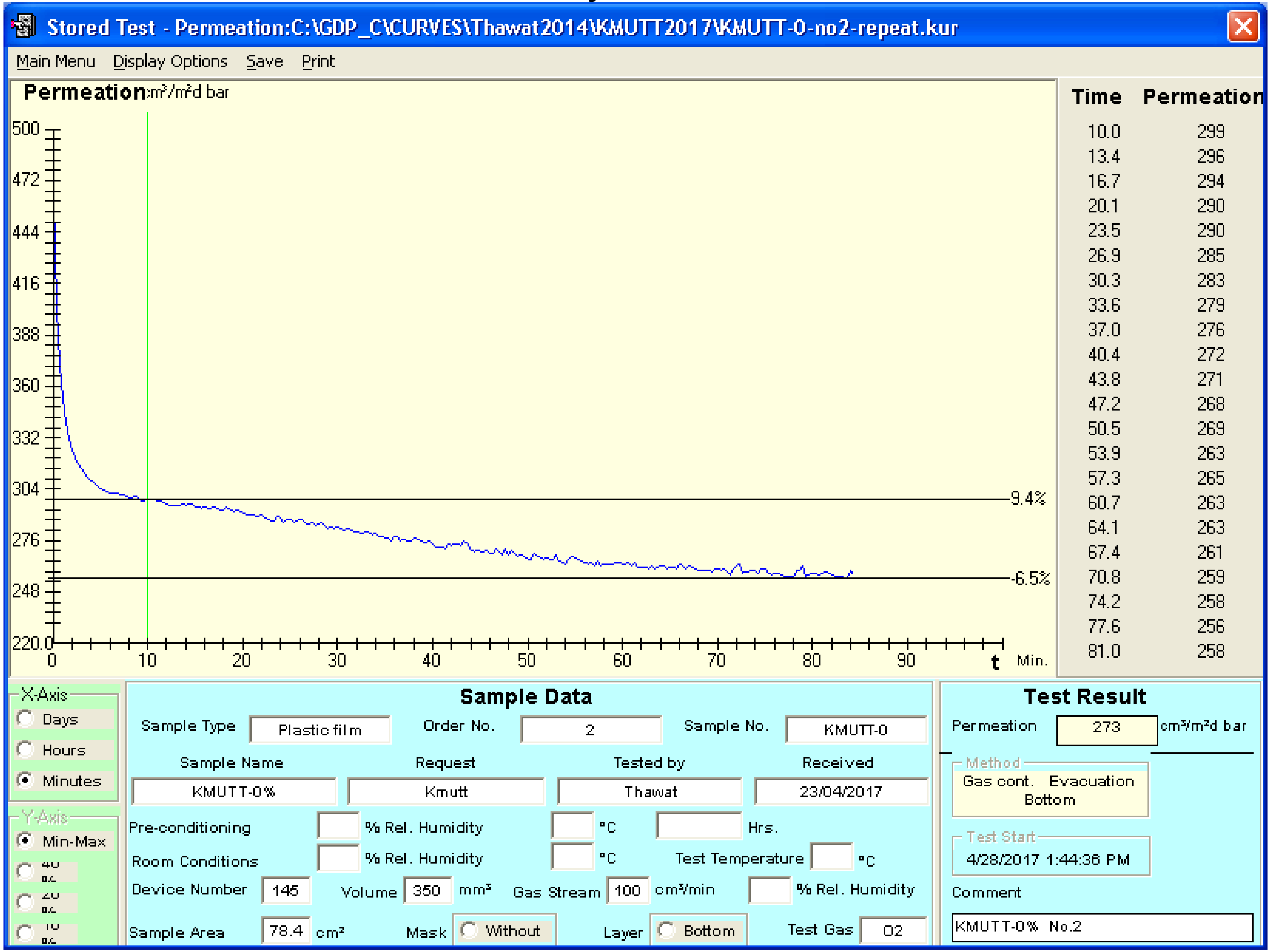

Surlyn film#3

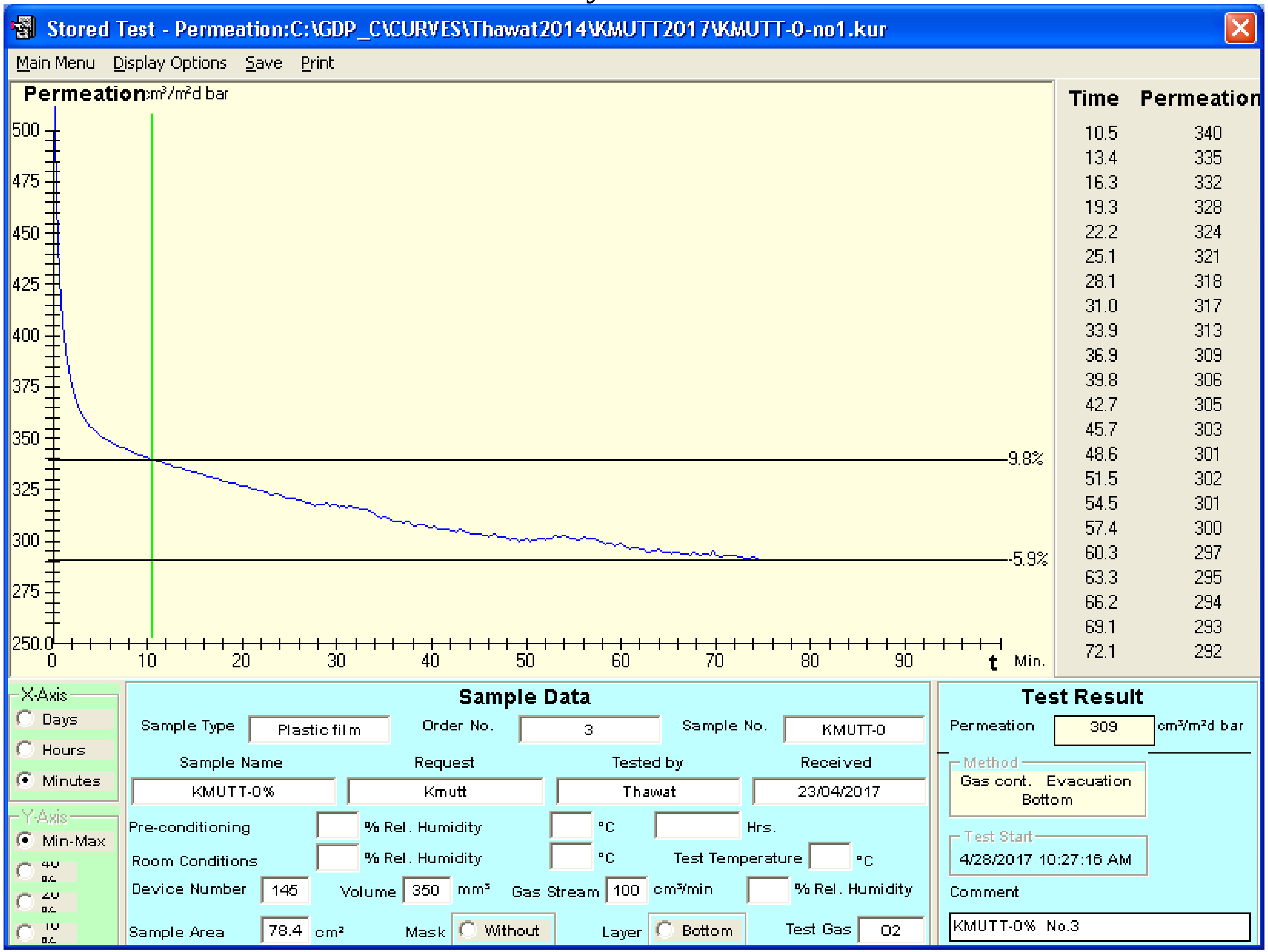

Surlyn/MFC-0.8(MB) #1

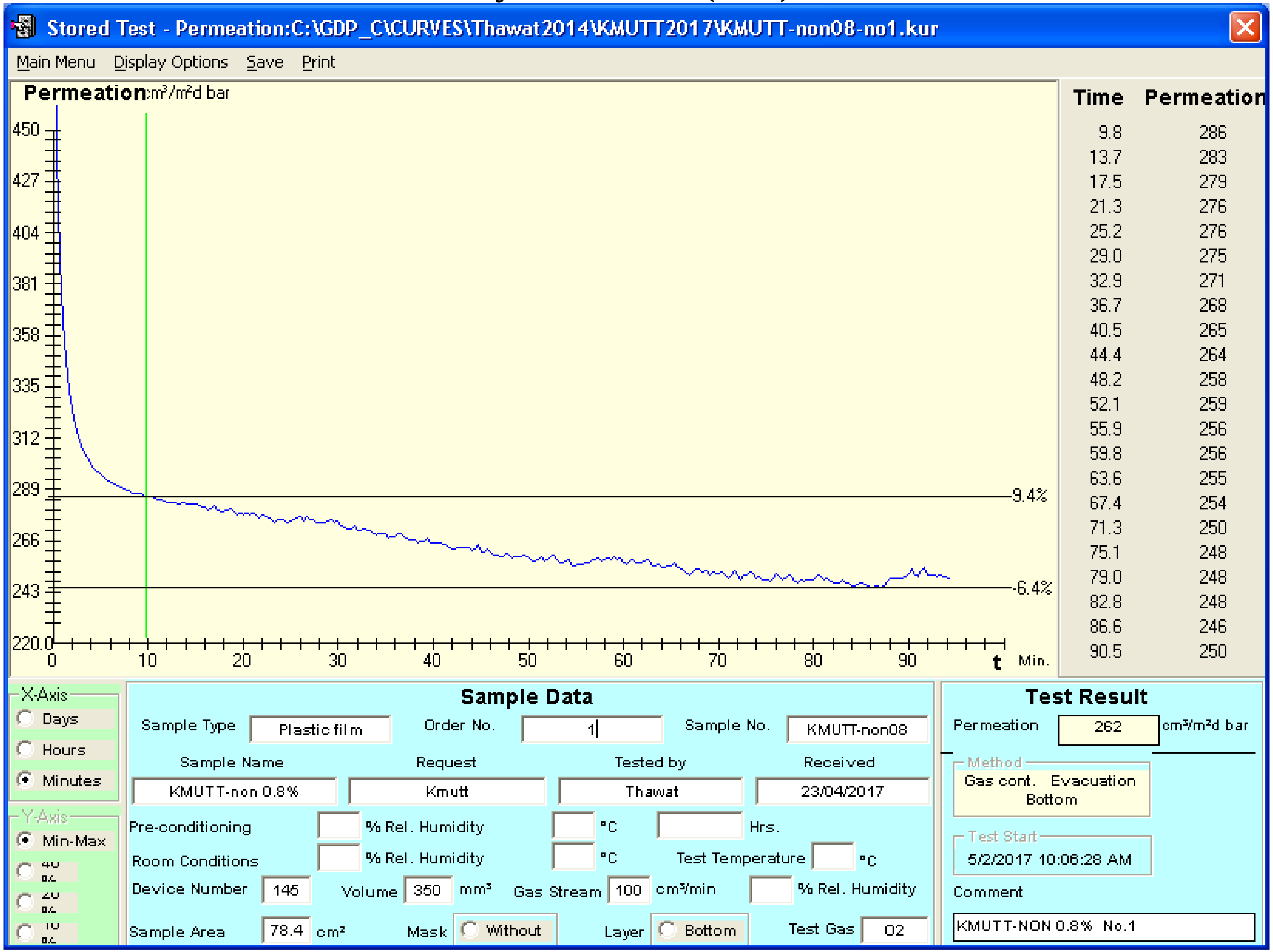

Surlyn/MFC-0.8(MB) #2

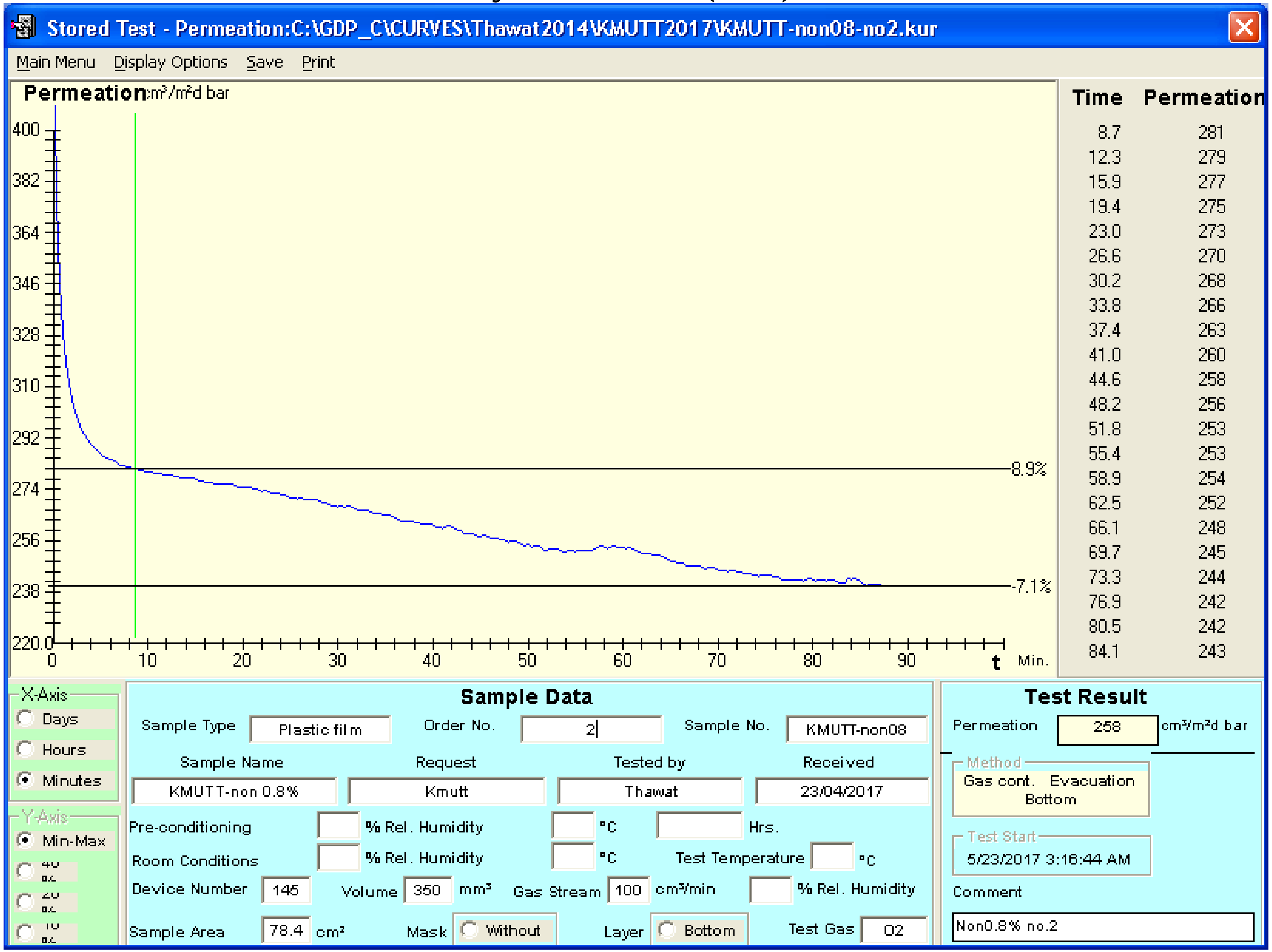

Surlyn/MFC-0.8(MB) #3

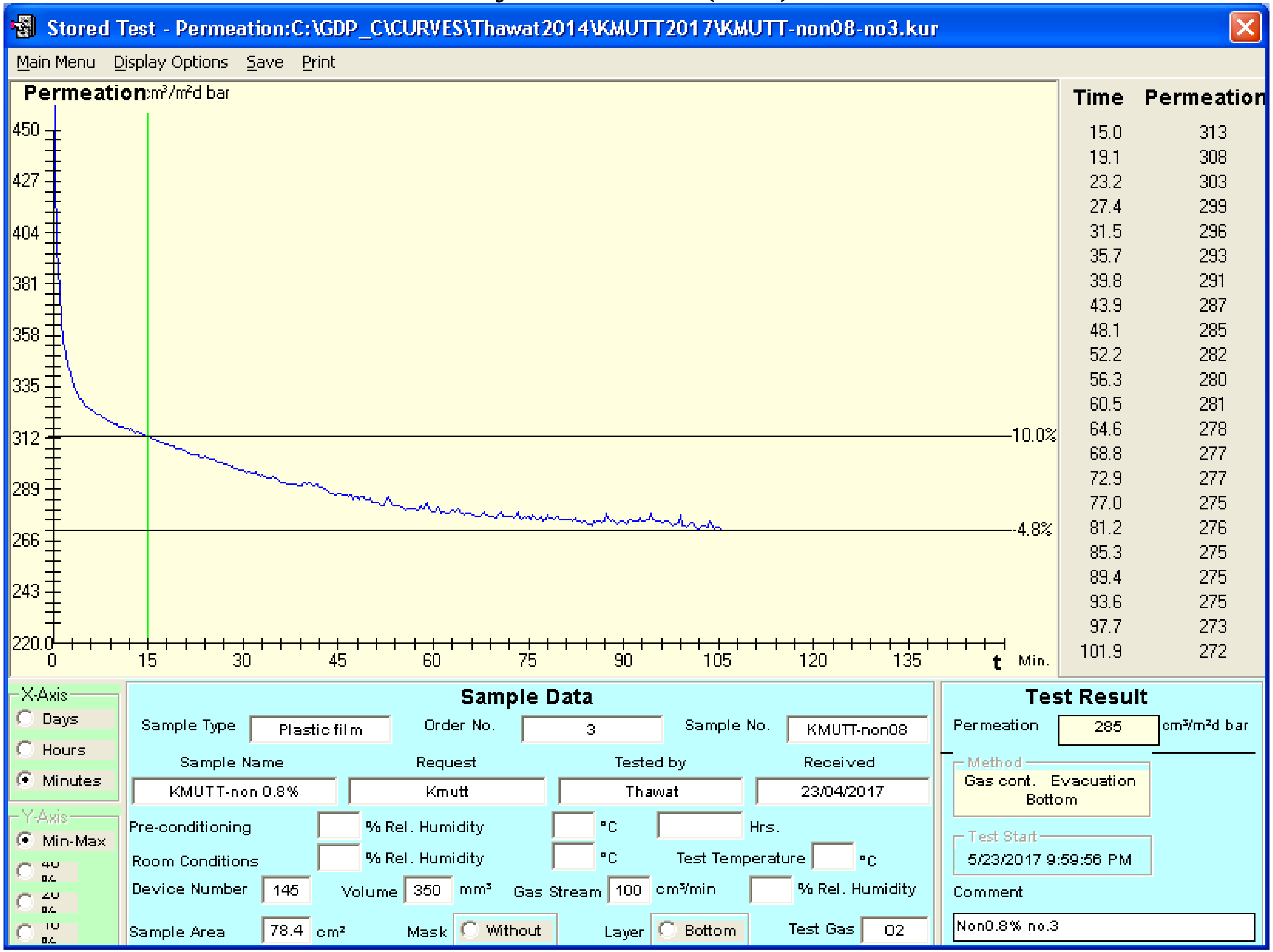

Surlyn/MFC-1.0(MB) #1

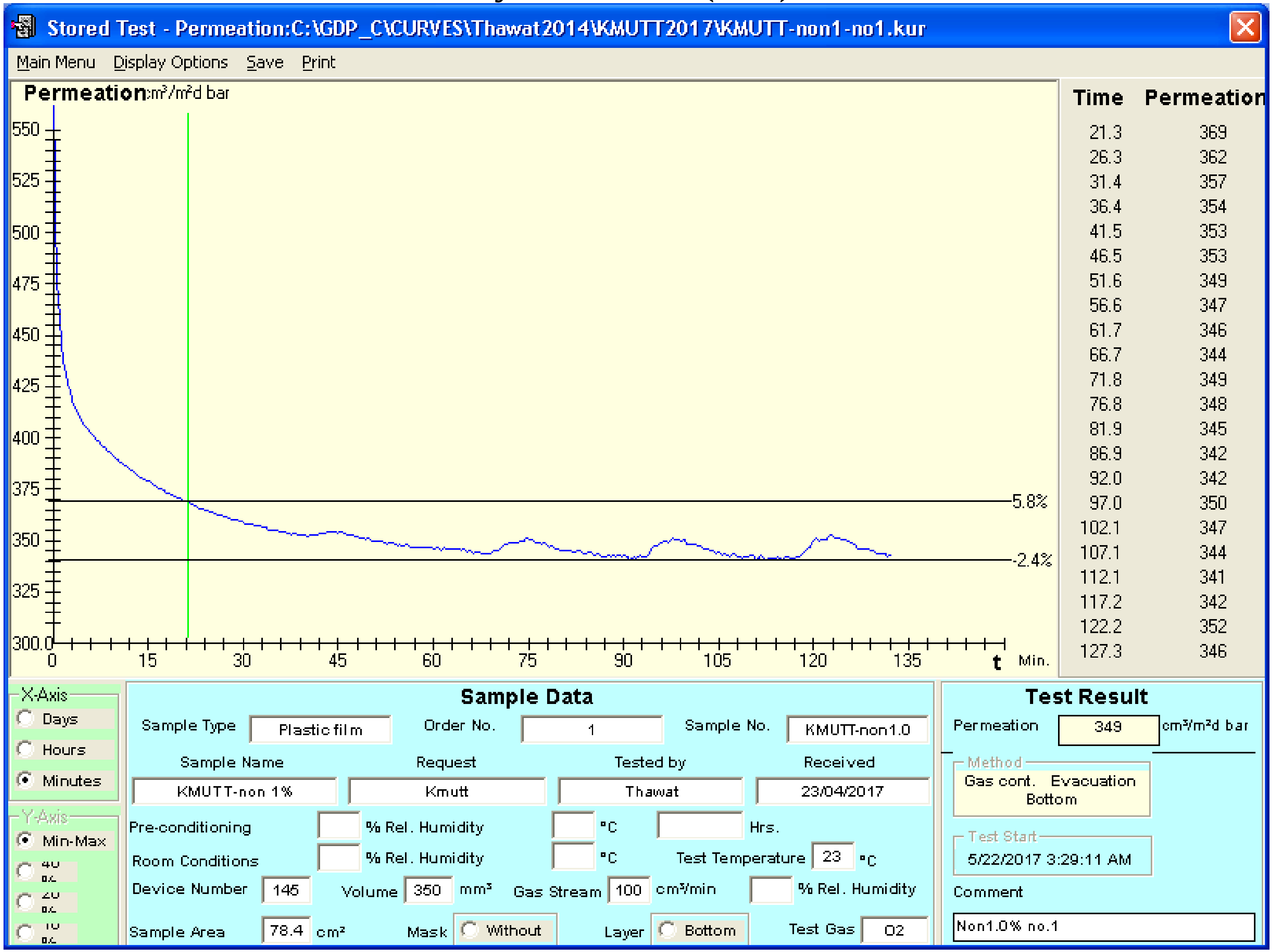

Surlyn/MFC-1.0(MB) #2

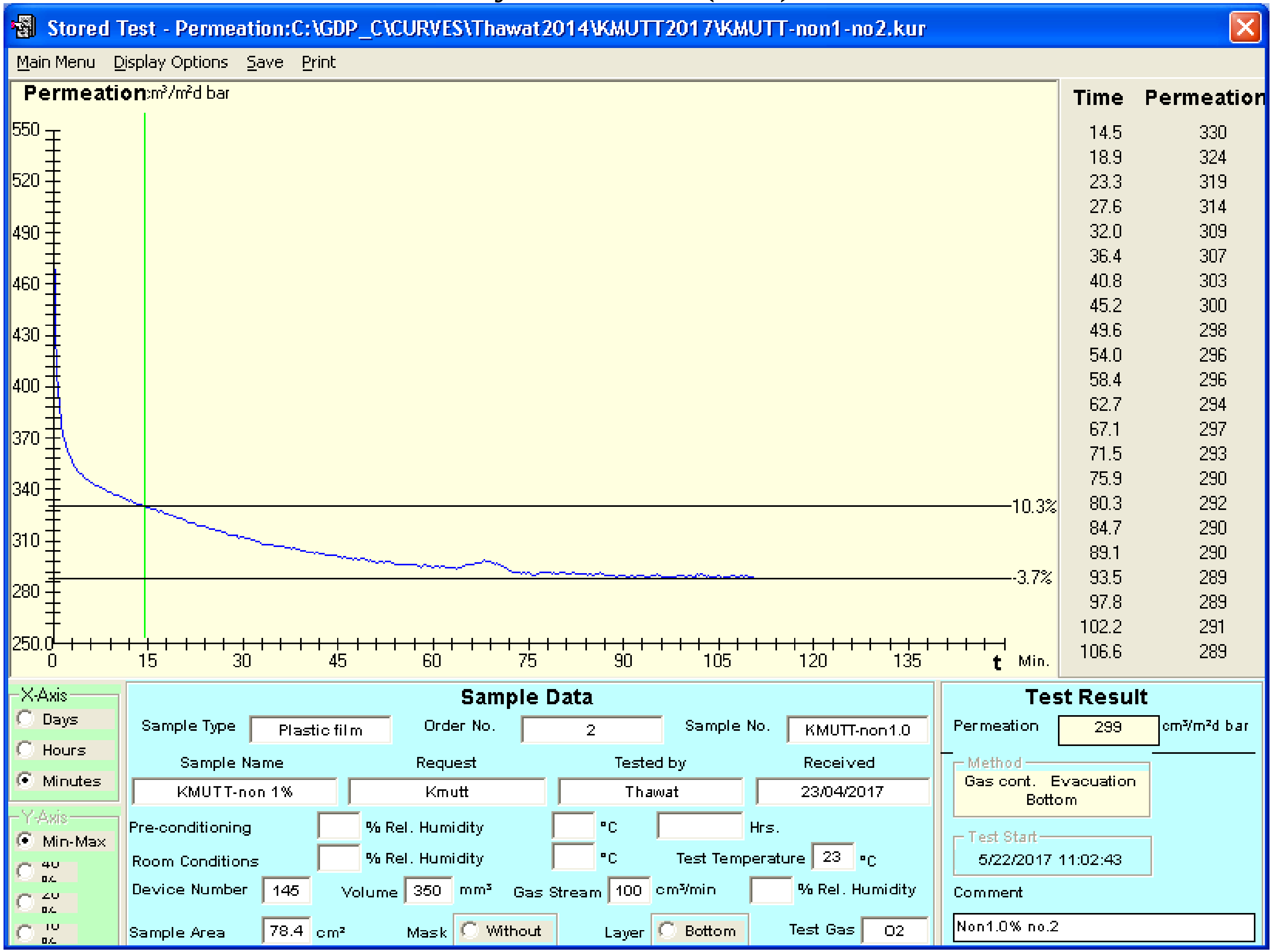

Surlyn/MFC-1.0(MB) #3

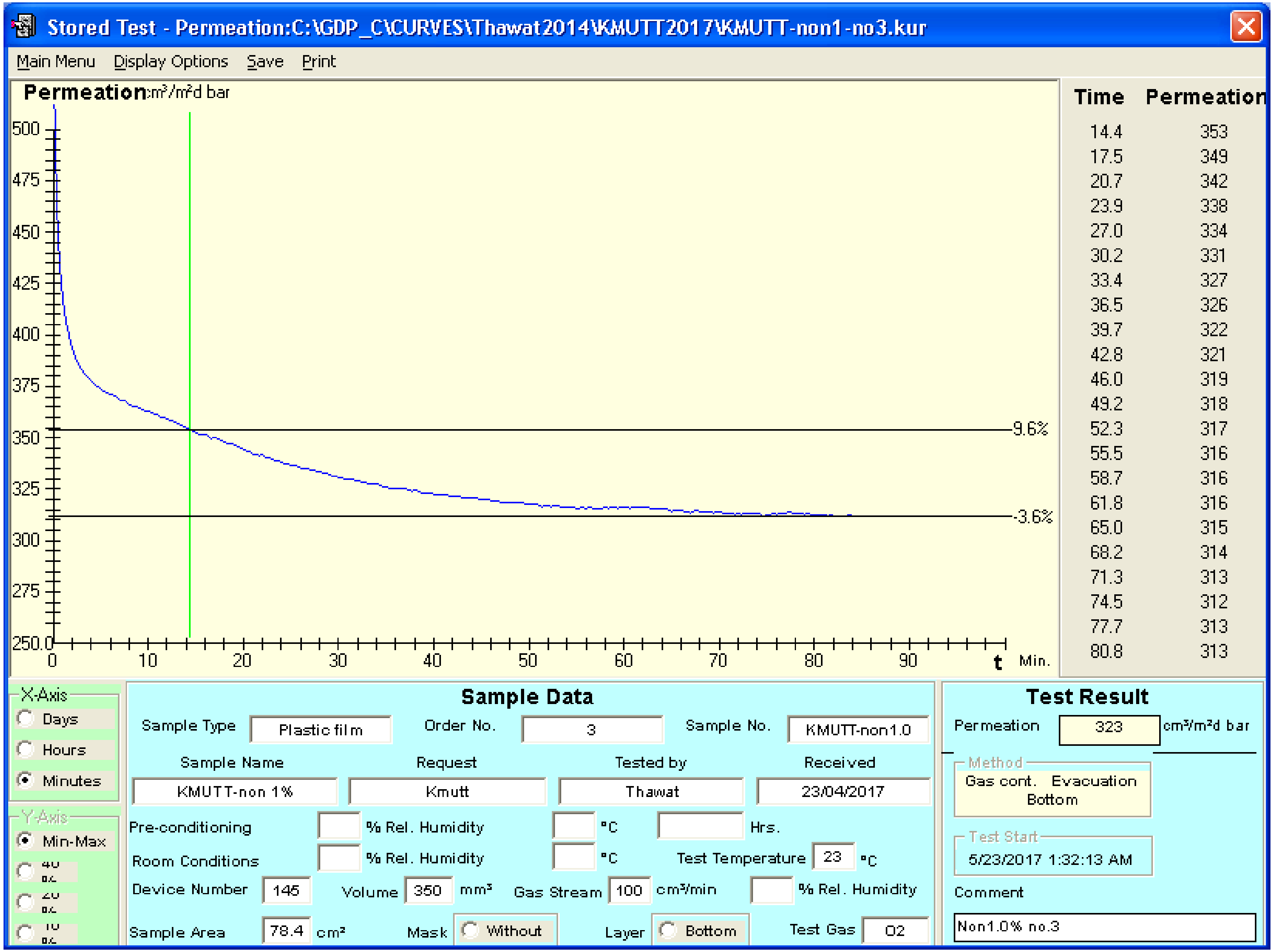

Surlyn/MFC-3.0(MB) #1

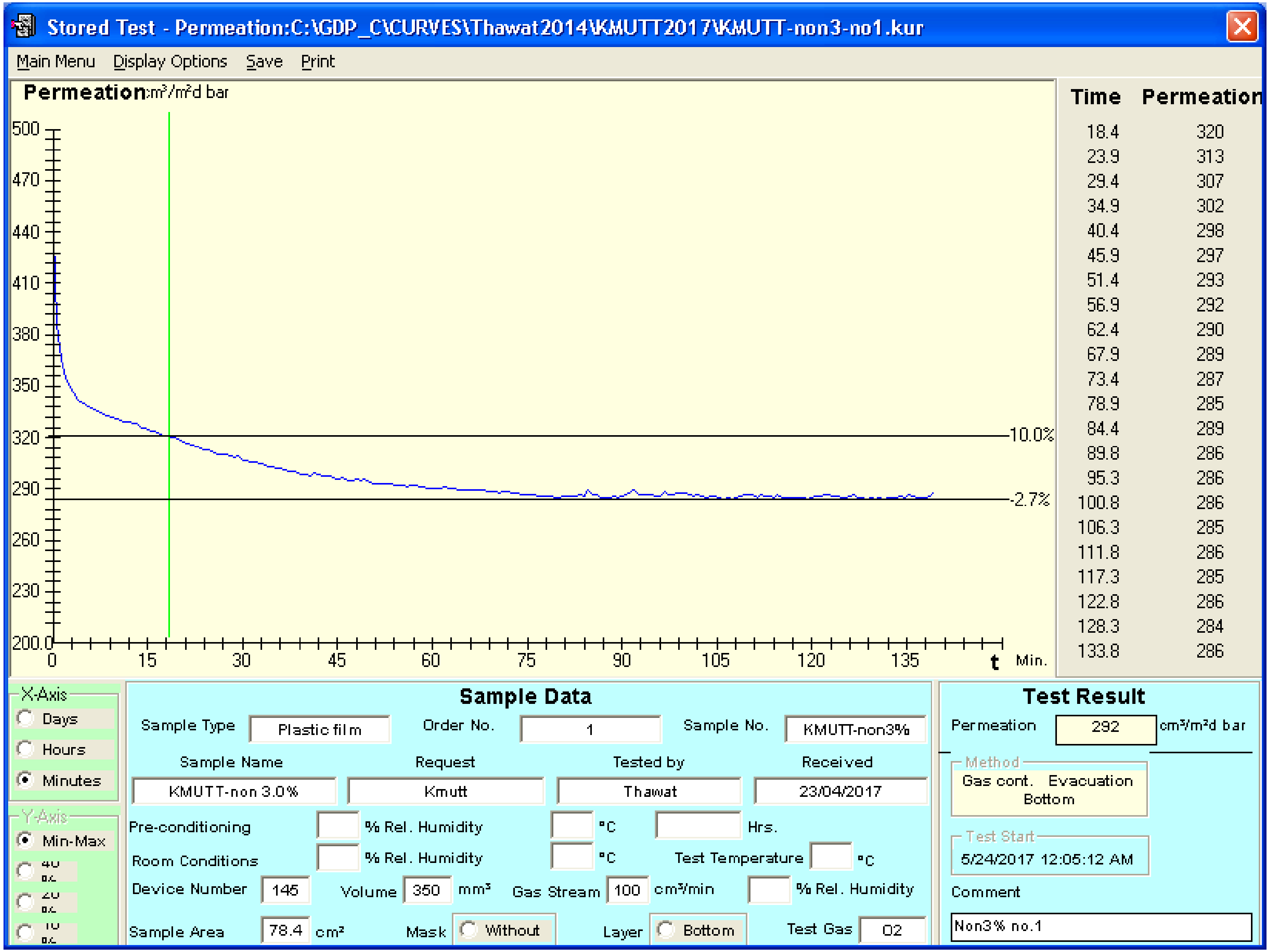

Surlyn/MFC-3.0(MB) #2

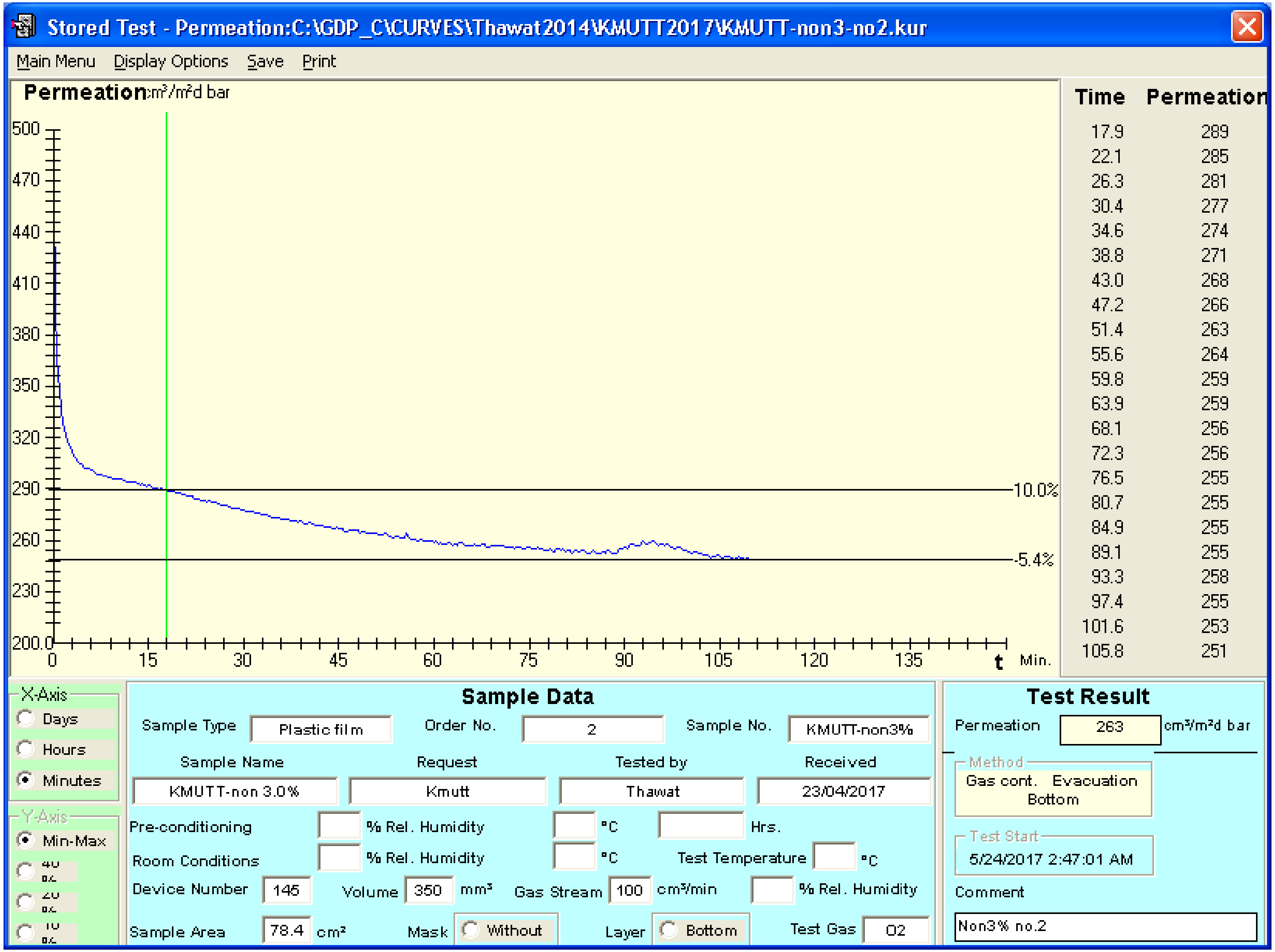

Surlyn/MFC-3.0(MB) #3

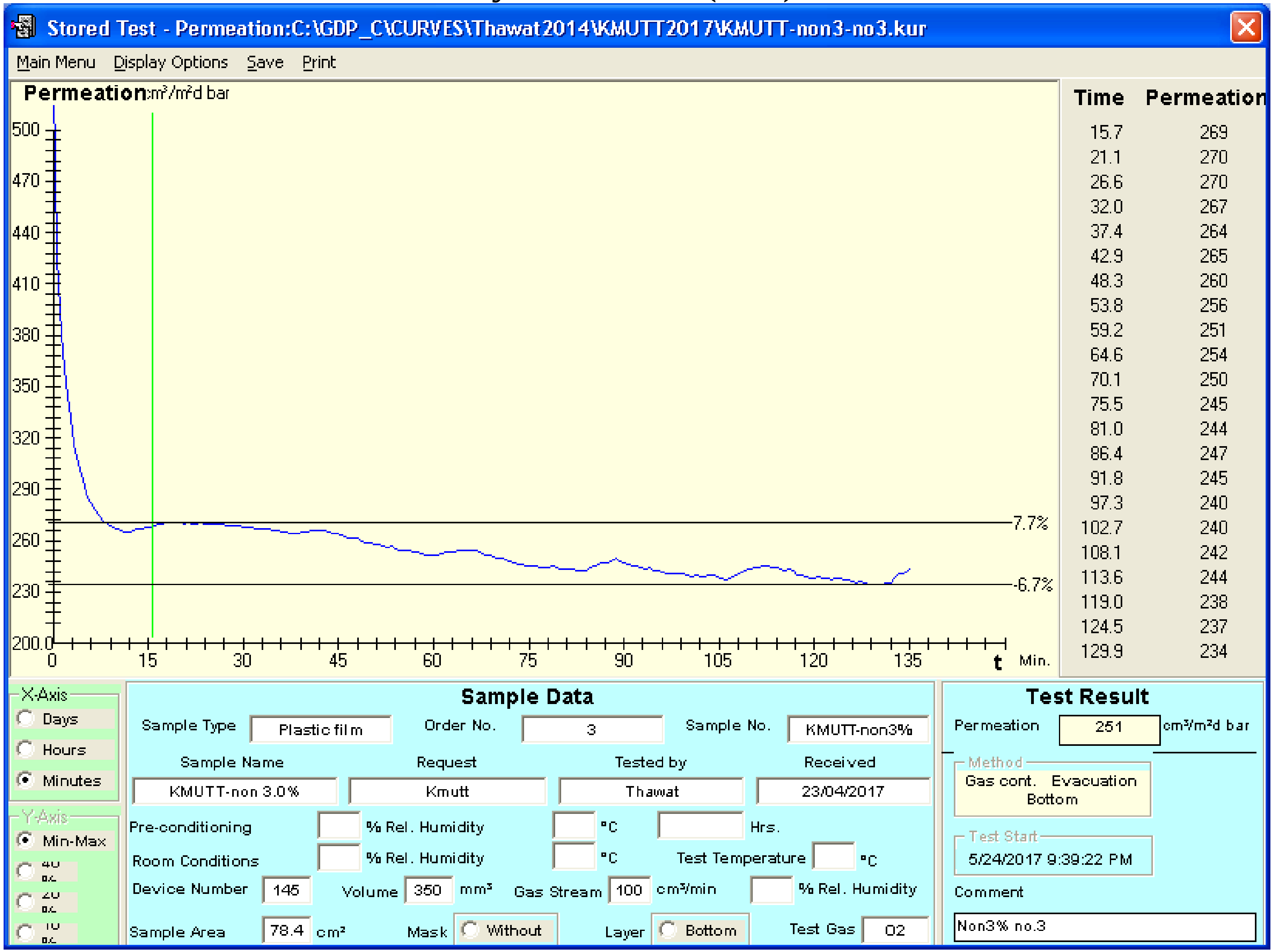

Surlyn/m-MFC-0.8(MB) #1

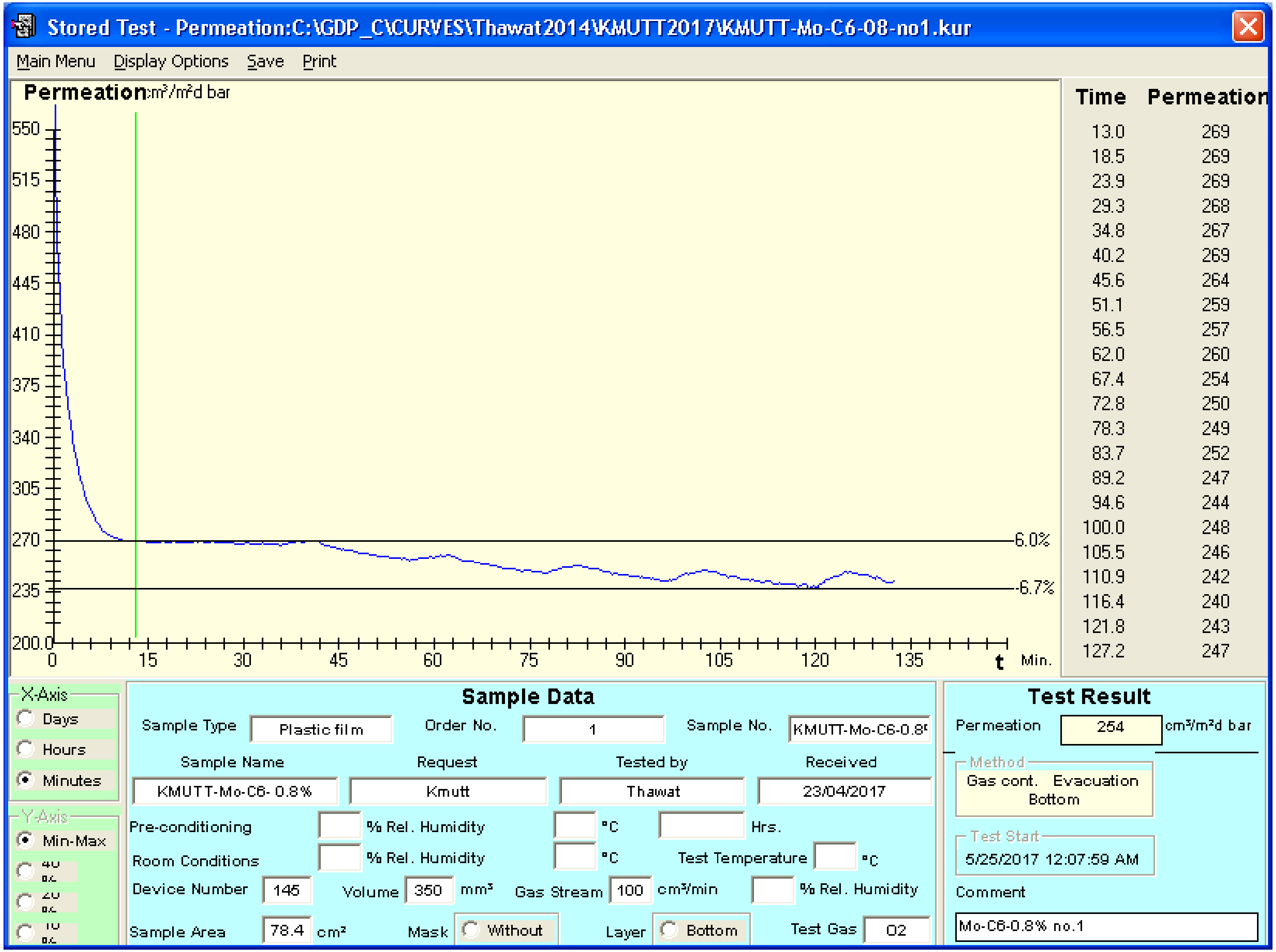

Surlyn/m-MFC-0.8(MB) #2

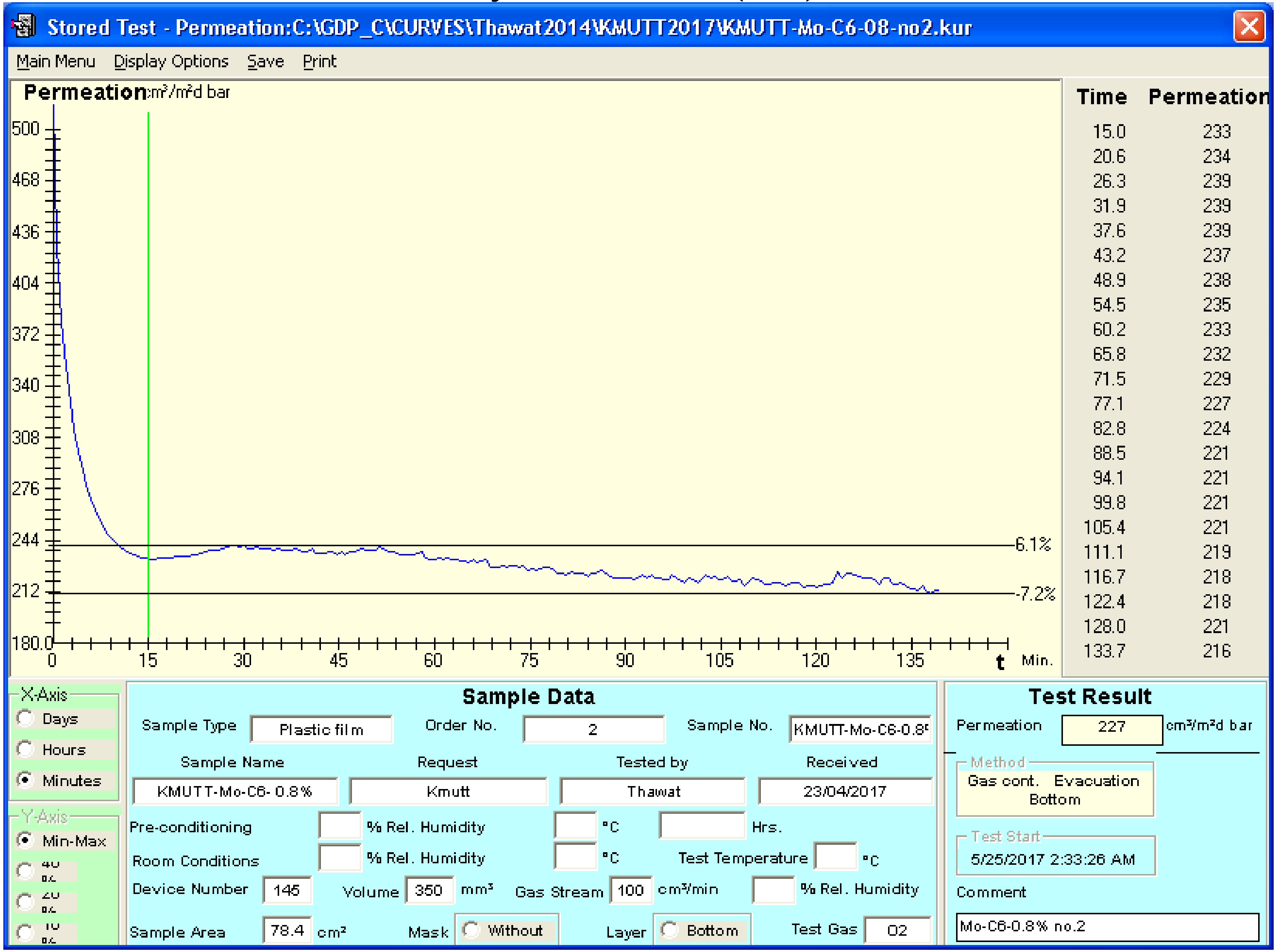

Surlyn/m-MFC-0.8(MB) #3

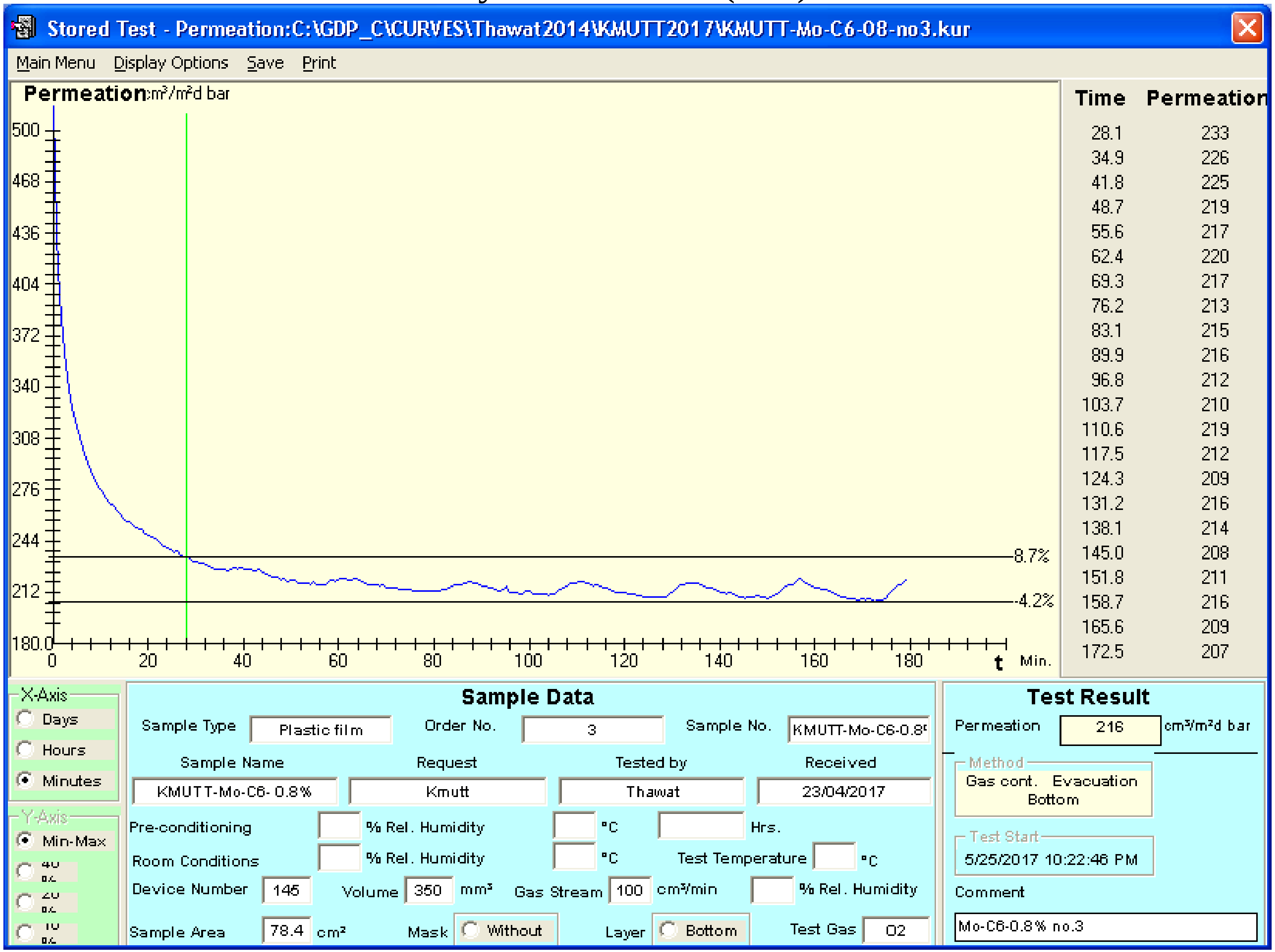

Surlyn/m-MFC-1.0(MB) #1

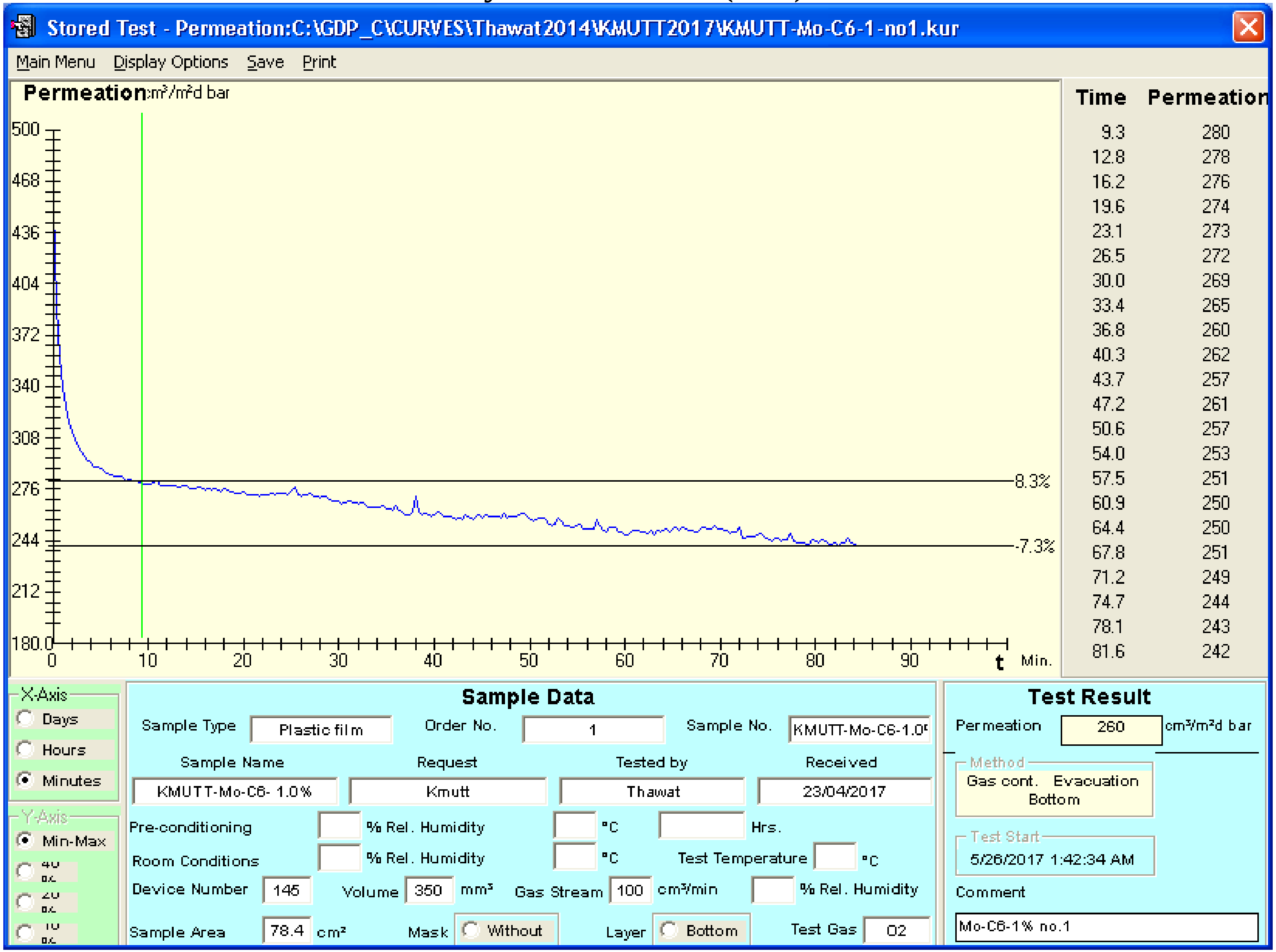

Surlyn/m-MFC-1.0(MB) #2

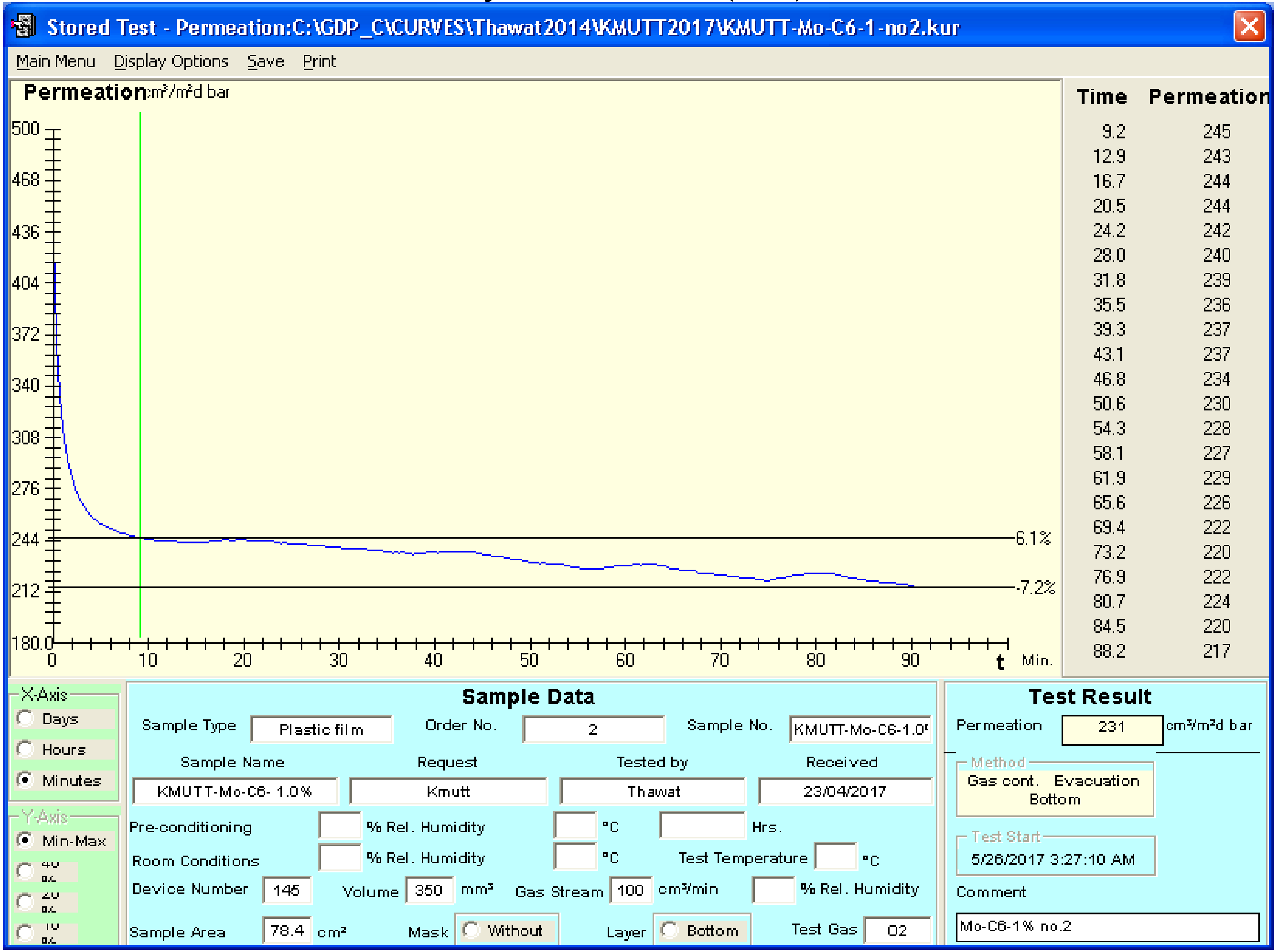

Surlyn/m-MFC-1.0(MB) #3

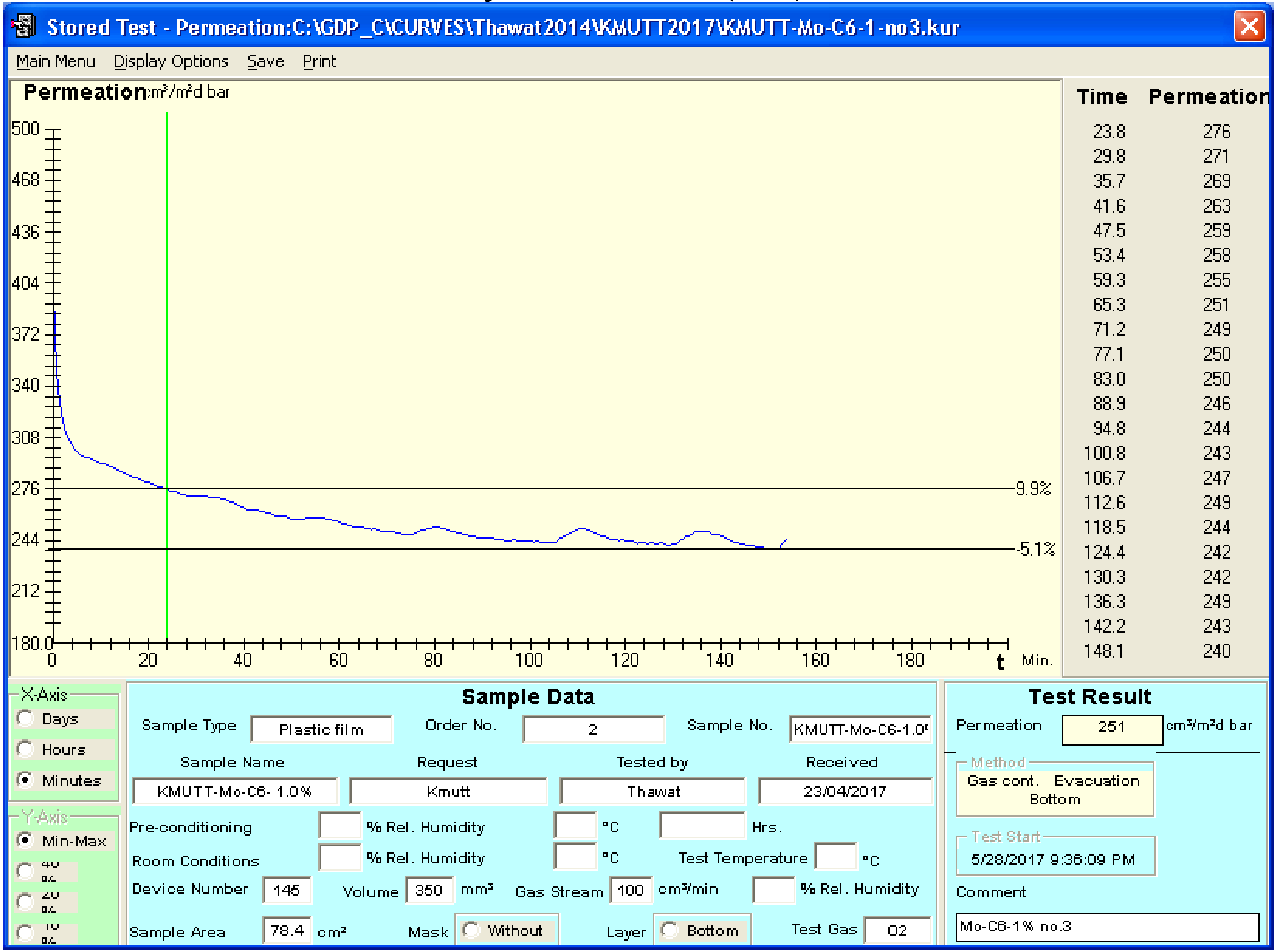

Surlyn/m-MFC-3.0(MB) #1

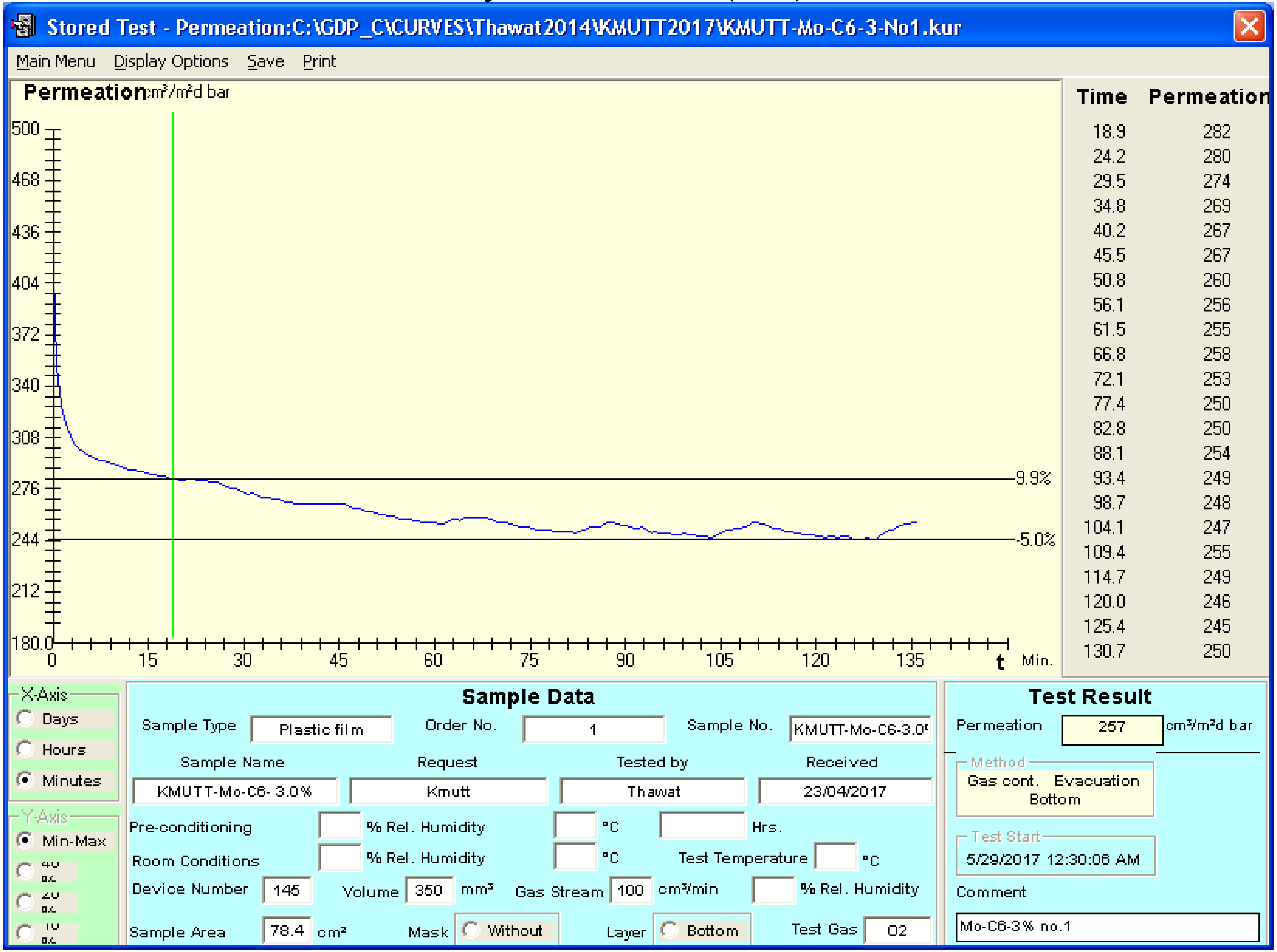

Surlyn/m-MFC-3.0(MB) #2

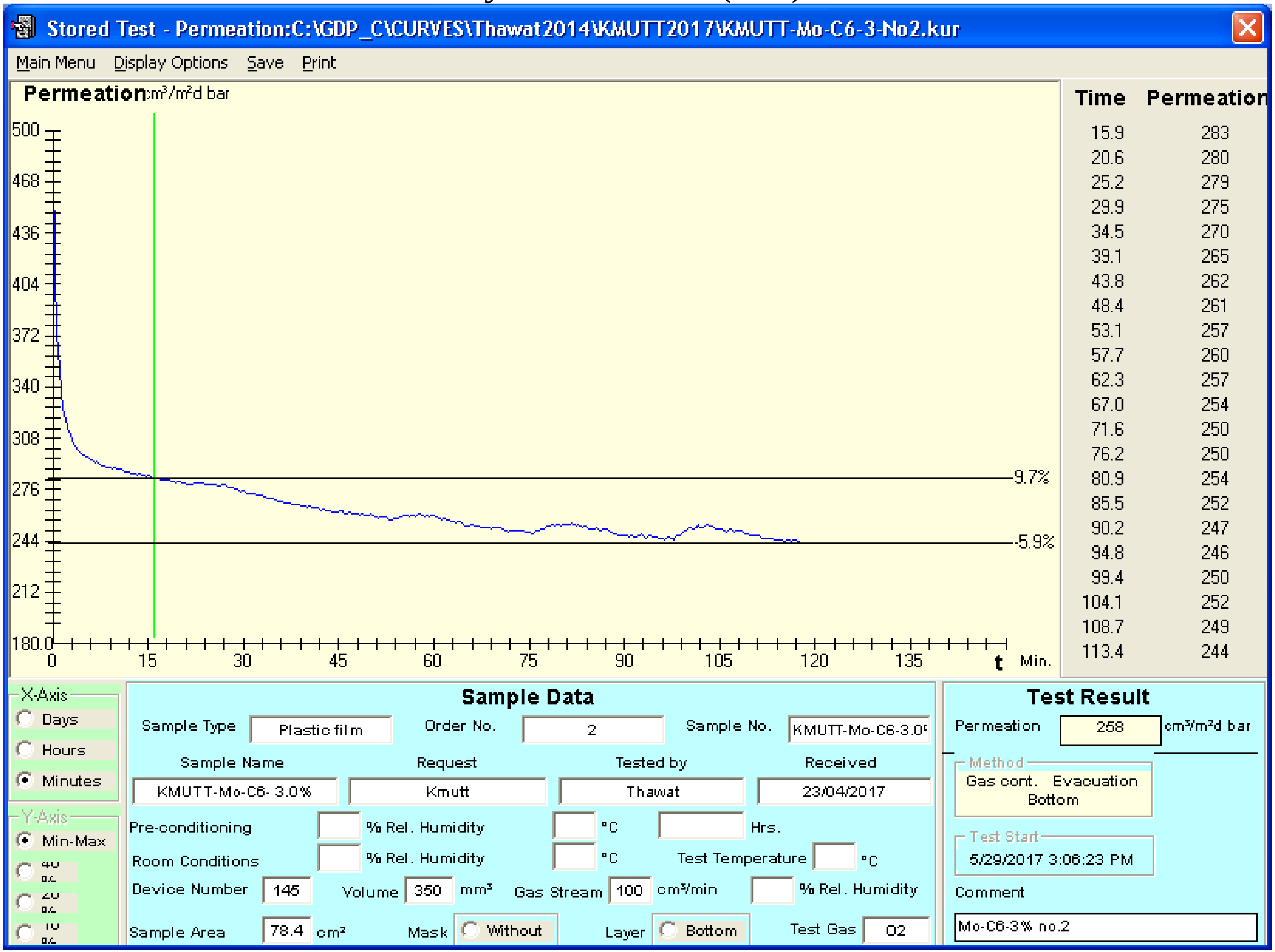

Surlyn/m-MFC-3.0(MB) #3

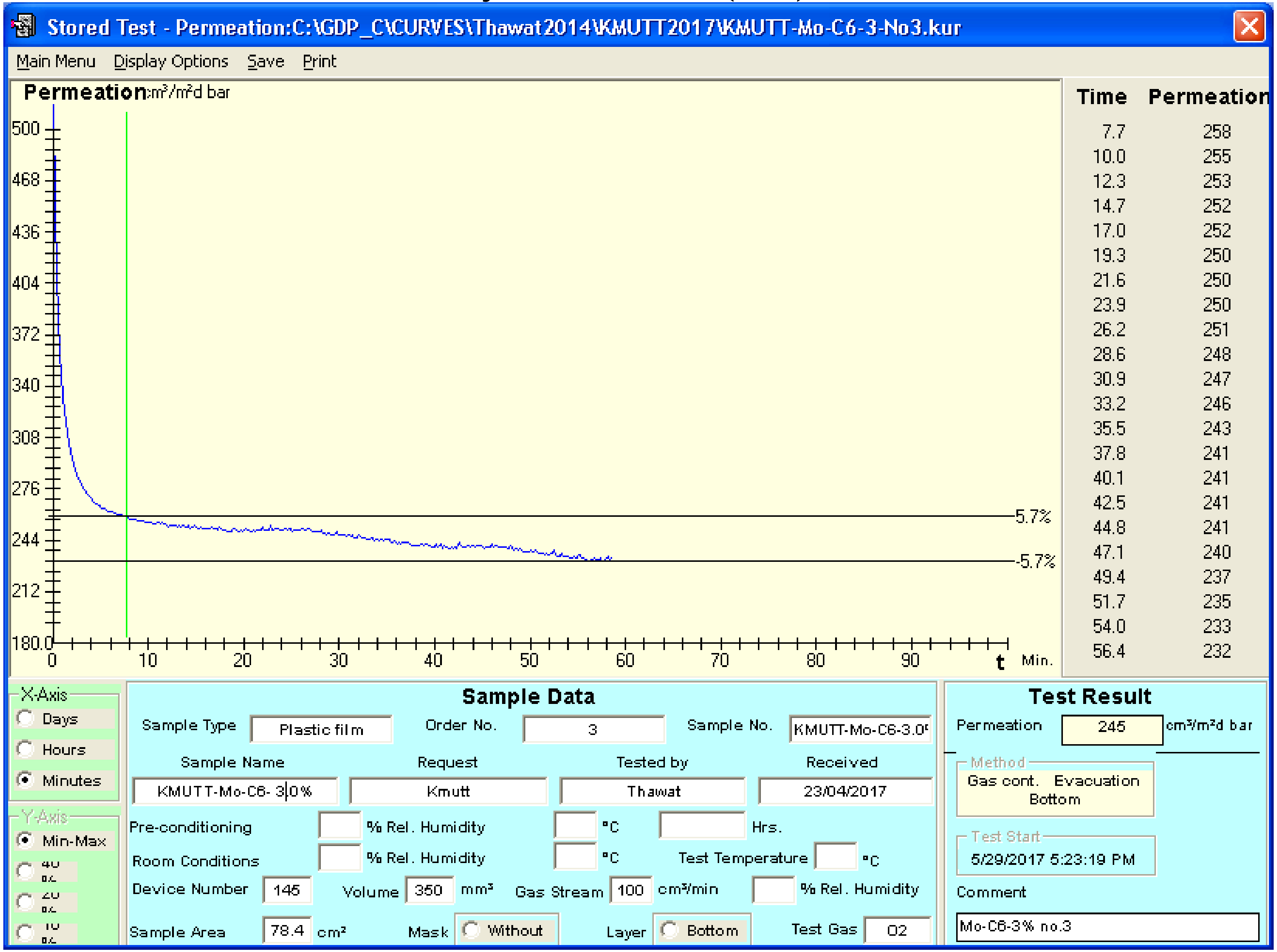

Supplement: DSC thermograms of the Surlyn-MFC composite films [file rsos170792supp1.pdf]
